# Lineages, Virulence Gene Associated and Integrons among Extended Spectrum β-Lactamase (ESBL) and CMY-2 Producing *Enterobacteriaceae* from Bovine Mastitis, in Tunisia

**DOI:** 10.3390/pathogens11080948

**Published:** 2022-08-22

**Authors:** Ahlem Jouini, Amira Klibi, Souhir Kmiha, Safa Hamrouni, Abdeljelil Ghram, Abderrazak Maaroufi

**Affiliations:** Laboratory of Epidemiology and Veterinary Microbiology, Group of Bacteriology and Biotechnology Development, Institute Pasteur of Tunis, University of Tunis El Manar, Tunis 1002, Tunisia

**Keywords:** ESBL, clonal lineages, p(AmpC), mastitis, pathogenic bacteria, Tunisia, virulence, integrons

## Abstract

Extended Spectrum Beta-Lactamase (ESBL) *Enterobacteriaceae* are becoming widespread enzymes in food-producing animals worldwide. *Escherichia coli* and *Klebseilla pneumoniae* are two of the most significant pathogens causing mastitis. Our study focused on the characterization of the genetic support of ESBL/pAmpC and antibiotic resistance mechanisms in cefotaxime-resistant (CTX^R^) and susceptible (CTX^S^) *Enterobacteriaceae* isolates, recovered from bovine mastitis in Tunisia, as well as the analyses of their clonal lineage and virulence-associated genes. The study was carried out on 17 ESBL/pAmpC *E. coli* and *K. pneumoniae* and 50 CTX^S^ *E. coli*. Detection of resistance genes and clonal diversity was performed by PCR amplification and sequencing. The following β-lactamase genes were detected: *bla*CTX-M-15 (*n* = 6), *bla*CTX-M-15 + *bla*OXA-1 (2), *bla* CTX-M-15 + *bla*OXA-1 + *bla*TEM-1b (2), *bla*CTX-M-15 + *bla*TEM-1b (4), *bla*CMY-2 (3). The MLST showed the following STs: ST405 (*n* = 4 strains); ST58 (*n* = 3); ST155 (*n* = 3); ST471 (*n* = 2); and ST101 (*n* = 2). ST399 (*n* = 1) and ST617 (*n* = 1) were identified in p(AmpC) *E. coli* producer strains. The phylogroups A and B1 were the most detected ones, followed by the pathogenic phylogroup B2 that harbored the shigatoxin genes *stx1*/*stx2*, associated with the *cnf*, *fimA*, and *aer* virulence factors. The *qnrA*/*qnrB*, *aac(6′)-Ib-cr* genes and integrons class 1 with different gene cassettes were detected amongst these CTX^R^/^S^ isolated strains. The presence of different genetic lineages, associated with resistance and virulence genes in pathogenic bacteria in dairy farms, may complicate antibiotic therapies and pose a potential risk to public health.

## 1. Introduction

Bovine Mastitis (BM) is one of the most common and costly diseases that affects dairy cattle worldwide [1]. It leads to loss of milk production, mandatory culling, and costly veterinary services [2]. *Enterobacteriaceae* species such as *Escherichia coli* and *klebseilla pneumoniae* are considered as significant etiological agents of bovine mastitis [3].

Mastitis due to *E. coli* varies significantly from mild to very severe and even fatal infection [4,5], which is associated with clinical signs [6]. Besides, *E. coli* has a zoonotic character and is involved in severe gastrointestinal or urinary tract infections in humans. Although their prevalence is low, *Klebsiella* infections in cattle appear to be particularly problematic due to their relatively long period of infection, leading to significant losses of milk production and increased mortality of affected cows [7]. Most *E. coli* infections associated with clinical mastitis are typically commensal; however, pathogenic variants have also been reported [8] and shigatoxigenic *E. coli* (STEC) are amongst the pathogenic variants inducing clinical mastitis [8]. Several studies elucidated the virulence determinants and reported shigatoxin-encoding genes (*stx1*, *stx2*) as being the most important virulence determinants in *E. coli* isolated from bovine mastitis [9]. The effect of the virulence factors (VFs) of *E. coli* on the severity of clinical mastitis in dairy cattle remains unclear [10].

Mastitis was reported as the most common consequence of antimicrobial administration in livestock [11]. Although, these drugs may also be used in a prophylactic manner, to prevent bacterial infection or promote livestock growth, this trend can adversely affect the World Health Organization (WHO) ‘One Health’ policy because of the ease with which Gram-negative bacteria (GNB) harbor multidrug resistance genes and can zoonotically move from animals to humans and vice versa [12]. The overuse of the extended spectrum cephalosporins causes the emergence of extended spectrum beta –lactamases (ESBLs) and/or plasmid-mediated AmpC (pAmpC) producing *Enterobacteriaceae* [13]. Based on the diversity of ESBL enzymes among *E. coli/K. pneumoniae* isolates from bovine mastitis, CTX-M-1 and CTX-M-14-producing *E. coli* are broadly disseminated in bovine mastitis cases in Europe [14]. On the contrary, CTX-M-15 was the most prevalent ESBL type found in *E. coli* mastitis in Asia [13,15]. Furthermore, *Enterobacteriaceae* producing p(AmpC) β-lactamases are more alarming than those possessing ESBLs because they are poorly inhibited by the commercially available β-lactamase inhibitors (i.e., clavulanate, sulbactam, and tazobactam) [16]. In particular, the CMY-2 types represent the most commonly encountered p(AmpC) β-lactamases identified in *E. coli* and other *Enterobacteriaceae* of human origin [17]. However, this β-lactamase variant was identified among commensally *E. coli* from healthy and diseased animals in Tunisia [18,19].

There are few reports describing the genetic features of *E. coli* and *K. pneumoniae* strains of bovine mastitis origin; the observed data being very heterogeneous and the genetic diversity remaining unclear.

This study aims to characterize the genetic support of ESBL/pAmpC *Enterobacteriaceae* isolates and the study of antibiotic resistance mechanisms in cefotaxime-susceptible (CTX^S^) *E. coli* isolates recovered from bovine mastitis in Tunisia, as well as the analysis of their clonal lineage and their virulence-associated genes. 

## 2. Results

### 2.1. Antibiotic Resistance Rates for ESBL/pAmpC Enterobacteriaceae Isolates

The occurrences of antibiotic resistance of 14 ESBL *Enterobacteriaceae* (ESBL-*EB*) (10 *E. coli*, 4 *K. pneumoniaeae* isolates) and 3 cefotaxime-resistant *E. coli* isolates are presented in Table 1.

The analysis of the antibiotic susceptibility test for all tested isolates showed high rates of resistance for tetracycline (82.35%), sulfonamides (76.5%), and nalidixic acid (64.5%). Moderate to low rates of resistance were observed for trimethoprim/sulfamethoxazole (47%), streptomycin (23.5%), ciprofloxacin (29.4%), and chloramphenicol (6%); no resistant isolate being detected for imipenem or ertapenem. Ten isolates showed multi-drug resistance phenotypes, including resistance to at least three families of antimicrobial agents. According to double disc synergy test (DDST), three *E. coli* CTX^R^ strains were AmpC producers and presented resistance to cefoxitin and amoxicillin-clavulanic acid.

### 2.2. Genetic Support of ESBL/p(AmpC) Enterobacteriaceae Isolates

**Figure 1:**
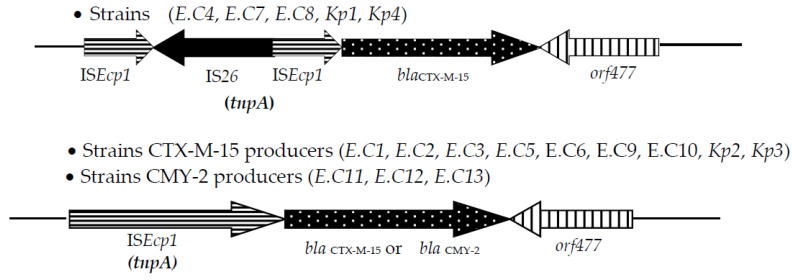
Genetic environments of *bla*_CTX-M_ and *bla*_CMY_ genes detected among our ESBL-positive *E. coli* and *K. pneumoniae* strains.

As shown in Table 1, the amplification and sequencing of β-lactamase genes of 14 (ESBL- *EB)* and 3 p(AmpC) producers of *E. coli* isolates revealed that all the tested ESBL *E. coli* or *K. pneumoniae* strains carried *bla*_CTXM-15_ or co-associated to *bla*oxa_-1_ + *bla*_TEM1-b_ (*n* = 2 strains), *bla*oxa_-1_ (*n* = 2), and *bla*_TEM1-b_ (*n* = 4); *bla*_CMY-2_ being detected in the three p(AmpC) phenotype *E. coli* strains. The regions surrounding both *bla*_CTX-M-15_ and *bla*_CMY-2_ genes were analyzed by PCR. The *orf*477 sequence was detected downstream of *bla*_CTX-M-15_ and *bla*_CMY-2_ in all ESBL and p(AmpC) producer strains. Nevertheless, ISE*cp1* sequence was identified upstream of *bla*_CTX-M-15_/*bla*_CMY-2_ gene in 12 isolates. Interestingly, the IS*26* flanked a partially truncated ISE*cp1* element in five *bla*_CTX-M-15_ positive *E. coli* isolates (Figure 1). The sequences of the amplicons of ESBL/p(AmpC) gene detected in this study were presented in FASTA form as a Supplementary file.

### 2.3. Integrons and Resistance Mechanisms to Non β-Lactam Antimicrobials Agents of ESBL-EB and p(AmpC)-Producer E. coli Strains

**Figure 2:**
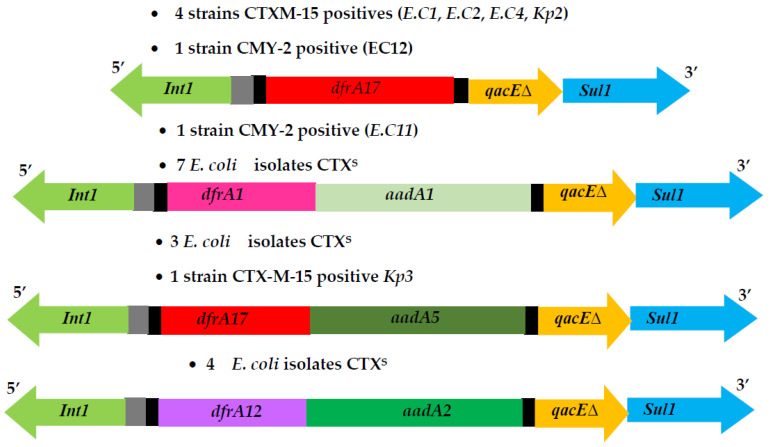
Integrons class 1 structures in ESBL *Enterobacteriaceae*, p(AmpC) *E. coli* producers and *E. coli* CTX^S^ isolates.

Table 1 presents antibiotic resistance phenotypes and genotypes of different (ESBL-*Eb*) and p(AmpC) *E. coli* strains. Class 1 integrons were detected in 7 out of 14 (ESBL-*EB*) and 3 p(AmpC)-producer *E. coli* isolates (41.17%); class 2 integrons were not identified in this collection. Three different arrangements were detected in variable regions of class 1 integrons: (1) *dfrA17* confers resistance to trimethoprim in five strains; (2) *dfrA1 + aadA1* were involved in streptomycin and trimethoprim resistance, respectively, in one strain; (3) *dfrA17 + aadA5* (1 strain). Figure 2 illustrated the structures of integrons class 1 in ESBL/p (AmpC) *Enterobacteriaceae* strains and CTX^S^ *E. coli* isolates.

The sequences of the amplicons of the variable region of integrons class 1 for each gene cassette detected in this study are presented in FASTA form as a Supplementary file.

Different resistance genes to non β-lactam antibiotics situated outside the integrons were observed in all tested strains: the *tet(A)* and *tet(B)* genes were detected in five and nine tetracycline resistance (ESBL-*Eb*) and p(AmpC) *E. coli* strains, respectively. Quinolones resistance was conferred by *aac(6′)–Ib-cr* gene in three *E. coli* and 1 *K. pneumonie* strains, by *qnrA* in three *E. coli* including one CMY-2 producer strain, and by *qnrB* gene in two *K. pneumoniae* strains. Different variants of *sul* genes were observed in 11 sulfonamide resistance strains: *sul2* + *sul3* (*n* = 3), *sul1* + *sul2* (*n* = 2), *sul2* (*n* = 5), and *sul3* (*n* = 1). The streptomycin resistance was conferred by *strA* gene in two strains.

### 2.4. Molecular Typing, Virulence Factors and Phylotyping of ESBL–EB and p(AmpC)-Producer E. coli Strains

The analyses of multilocus-sequence typing (MLST) showed seven sequence types (STs); the most frequent being ST405 (*n* = 4 strains); ST58 (*n* = 3); ST155 (*n* = 3); ST471 (*n* = 2); and ST101 (*n* = 2). The remaining two STs, ST399/*n* = 1 and ST617/*n* = 1, were identified in p(AmpC) *E. coli* producer strains. The sequences of the amplicons of the seven housekeeping genes of *E. coli* and *K. pneumoniae* for each sequence type detected in this study are presented in FASTA form as a Supplementary file.

The phylogeny analyses revealed that our isolates belong to phylogroups A (six isolates), B1(four isolates), D (one isolate), and B2 (two isolates). For virulence determinant factors, only two strains assigned to the phylogroup B2, harbored two associations out of three virulence factors (*cnf1*, *stx2*, *aer*) and (*aer*, *fimA*, *stx2*) among the genes investigated. In addition, the ESBL *E. coli* producer isolates assigned to the phylogroup D harbored only the *aer* and *stx2* virulence factors. The virulence factors *stx1* and *stx2* were detected in three *E. coli* strains and were characterized as shigatoxigenic *E. coli* (STEC).

### 2.5. Antibiotic Resistance Rates for CTXS E. coli Isolates

Table 2 presents antibiotic resistance phenotypes of 50 *E. coli* isolates susceptible to cefotaxime. Amongst this collection, 28 *E. coli* isolates were resistant to at least three antibiotic families. The highest antimicrobial resistance phenotype of 50 *E. coli* was observed for ampicillin, ticarcillin, and tetracycline (78%, 66%, and 78%, respectively). For sulfonamides, streptomycin, and trimethoprim-sulfamethoxazole, the percentage of resistance was 52%, 40%, and 40%, respectively. Whilst low resistance has been detected to nalidixic acid, ciprofloxacin (4%), cefoxitin (10%), and chloramphenicol (6%), no isolate was resistant to carbapenem or aminoglycoside families (Figure S1).

In addition, similar different association of phenotype resistance were observed in our *E. coli* isolate collections from different farms and samples: [TET][AMP][SXT][TIC][SUL][STR] (*n* = 12); [TET][AMP][SXT][TIC][SUL] (*n* = 7); [AMP][TIC][NAL][CIP] (*n* = 2) and [TET][AMP][TIC] (*n* = 6).

### 2.6. Genotypic Features of CTXS E. coli Isolates

The resistance genes detected among our 50 CTX^S^ *E. coli* isolates are shown in Table 2. The PCR amplification and sequencing of different β-lactamases resistance genes revealed that all ampicillin-resistant *E. coli* strains harbor *bla*_TEM-1b_ encoding TEM β-lactamase enzyme. Tetracycline resistance is conferred by *tet(A)* and *tet(B)* in 23 and 16 isolates, respectively. The *qnrA* gene was detected in five quinolone-resistant *E. coli* strains. Furthermore, the 24 isolates resistant to trimethoprim-sulfamethoxazol presented different types and combinations of *dfrA* and *aadA* genes (conferring resistance to trimetoprim and streptomycin). Some of them were identified inside the structure of class 1 integron in 14 strains, with the following gene cassette arrangements (number of strains): *dfrA1* + *aadA1* (7), *dfrA12* + *aadA2* (4), and *dfrA17* + *addA5* (3) (Table 2, Figure 2). Other different resistance genes were identified outside integrons [antibiotic/gene (number of isolates)]: sulfonamide/*sul2* (5); streptomycine/*strA* (8)/*strB* (1); chloramphenicol/*catA* (3); trimetoprim resistance being conferred by *dfrA1* (7) and *dfrA12* (1).

Phylogenetic group analyses by PCR amplification showed the prevalence of commensal phylogroup A (*n* = 21) followed by B1 (*n* = 18), while the virulent phylogroup B2 was detected in 11 *E. coli* strains. At least two to three virulence factors (*aer + cnf1*, f*imA* + *stx2*, stx2 + *cnf1, stx2 + aer + cnf1*) were detected in only the pathogenic phylogroup B2 *E. coli* isolates and six of them were characterized as shigatoxigenic *E. coli* (STEC) (Table 2).

## 3. Discussion

To the best of our knowledge, there is no published information regarding clonal lineages, virulence factors, and detection of p(AmpC) producing *E. coli* from milk of clinical mastitis in Tunisia. Only the study of Saidani et al. [20] reported some data on the antimicrobial resistance genes in ESBL *Enterobactericeae* recovered from bovine mastitis, in Tunisia; such type of data are very scarce in other African countries [21,22].

According to the report of Klibi et al. [23], ESBL-producing *E. coli* and *K. pneumonia* included in this study harbored *bla*_CTXM_ gene. For this, our study focused on the molecular characterization of the genetic support of ESBL/p(AmpC) *Enterobacteriaceae* isolates, the analyses of their phylogeny and clonal lineage, as well as the virulence factors revealed in 300 clinical mastitis milk samples collected from Tunisian farms. Furthermore, our study was also interested in characterizing the mechanism of antibiotic resistance, identifying the virulence factors and establishing the phylogeny of 50 cefotaxime susceptible *E. coli* isolates recovered from the same samples.

The sequencing and the analyses of CTXM gene of ESBL *Enterobacteriaceae* isolates producers showed the dominance of the variant *bla*_CTX-M-15_ β-lactamase in all ESBL strains, although CTX-M-15 has been frequently related to human ESBL-producing *Enterobacteriaceae* worldwide [24]. Recently, in Tunisia, it was reported in ESBL *E. coli* and *K. pneumoniae* strains from mastitis bovine milk [20]; in *E. coli* isolates recovered from diarrheic chickens [19]; and in feces of healthy poultry, sheep, goat, and calf [25].

Our findings are in accordance with previous studies that report ESBL-producing *E. coli* isolates from bovine mastitis cases in Egypt, Turkey, China, United Kingdom, and Germany [6,13,22,26,27]. Nevertheless, other studies have described that *bla*CTX-M-1 and *bla*_CTXM-14_ genes were the most detected from cows with mastitis in France [28] and *bla*_CTX-M-2_ type in Japan and Germany [27,29]. The emergence of *bla*_CTX-M-15_ type in *Enterobacteriaceae* isolates from cows was scary because ESBL *E. coli* and *K. pneumoniae* producers, especially this CTX-M type, are important causal agents of healthcare-oriented as well as community-based infections in humans [30]. The reason for such a frequent presence of ESBL-producing bacteria in cows with mastitis could be related to the use of β-lactam, especially cephalosporin β-lactam class in veterinary medicine [22]. On the other hand, the cause could generally be related to the location of ESBL genes on plasmids that may spread easily among commensal and pathogenic bacteria within herds and the environment. Furthermore, *bla*_CTX-M-15_ was associated to *bla*_OXA-1_ in four ESBL *E. coli*-producing strains. This association has been recently described in diarrheic poultry [19] and emerged in clinical ESBL *E. coli* strains in Tunisia [31,32].

It is important to highlight that the three CTX^R^ *E. coli* strains are p(AmpC) producers encoded by *bla*_CMY-2_. To the best of our knowledge, this appeared to be the first description of *bla*_CMY-2_ involved in bovine mastitis in Tunisia. This β-lactamase type was recently identified in *E. coli* isolated from diarrheic poultry [19]. However, its presence was rare in cows with bovine mastitis and only few reports identified *bla*_CMY_ from dairy cows with clinical mastitis in Lebanon [33], China [34], Taiwan [35], and Switzerland [36].

Antimicrobial susceptibility testing showed that 10 ESBL/p(AmpC) *Enterobacteriaceae* producers and 28 of CTX^S^ *E. coli* isolates are multidrug resistant to at least three antibiotics families and present resistance to β-lactam (ampicillin, Ticarcillin), tetracyclines, quinolones, sulfonamides, and trimethoprim-sulphamethoxazole and aminoglycosides. Different reports confirmed that ESBL-producing *E. coli* and *K. pneumoniae* isolates are multiresistant, independently from their origin such as cattle [20,22], poultry [11,19] or human [31,37]. This finding is in concordance with the veterinary treatment used in Tunisia and based on penicillin, cloxacillin, streptomycin, enrofloxacin, marbofloxacin, sulfonamide, trimethoprim, or colistin, which are the most used antibiotics to treat bovine mastitis alone or in combination [20]. In the present study, tetracycline and sulfonamide resistances of ESBL/p(AmpC) *Enterobacteriaceae* and CTX^S^ *E. coli* isolates are coded by *tet* and *sul* genes, respectively. These results were in agreement with previous reports from Tunisia, Egypt, and Lebanon [20,22,33]. Moreover, it seems that *tet*(A) gene is the most dominant in our collection, identified from bovine mastitis cases in Tunisia. This is in line with the recent report, which described the highest proportion of *tet*(A) in cattle with mastitis in Egypt [22].

In this study, a high frequency of quinolone resistance (64.5% for nalidixic acid) was detected in ESBL/p(AmpC) *Enterobacteriaceae* strains. This frequency is higher than that reported by the only study interested in ESBL-*EB* strains from bovine mastitis in Tunisia [20]. Similar findings documented a high quinolone resistance rate in Egypt (85.7%) and in China (73.3%) [22,34]. Such correlation analysis between plasmid-mediated quinolone resistance (PMQR) and ESBL genes in ESBL-*EB* isolates showed that *aac(6′)-Ib-cr*, *qnrA*, and *bla*_CTX-M-15_ are significantly associated as reported by other studies [20,21,34], suggesting that the coexistence of quinolone resistance and ESBL genes has started to become epidemic in bovine mastitis in Tunisia. The detection of such genes was very interesting due to the spread of these resistance determinants between bacteria via plasmid mobility. Nevertheless, the quinolone resistance rate (10%) revealed in our CTX^S^ *E. coli* isolates was low and our results showed much lower rates than data reported from China and India [34,38] and even lower than those from developed countries such Finland and Canada where the use of quinolones in livestock has been limited [39].

It is important to highlight the presence of class1 intergrons in multidrug resistant *Enterobacteriaceae* with (*dfrA1, dfrA17, and dfrA12)* genes, conferring resistance to trimethoprim and (*aadA1*, *aadA2*, *aadA5*) encoding streptomycin resistance. To the best of our knowledge, the integrons were described in bovine mastitis for the first time in Tunisia. Antimicrobial resistance gene cassettes including integrons were detected in diarrheic chickens and poultry in Tunisia and Africa [11,19]. In fact, some studies were interested in integrons in *Enterobacteriaceae* from healthy bovine and with mastitis and only few recent reports have described integrons with different combinations of gene cassettes in *Enterococcus* and *S. aureus* in China [40,41]. These gene cassettes included in integrons remained largely unexplored in dairy cattle, suggesting the need for more studies on integrons and their circulation in livestock.

Phylogenetic typing analyses, carried out on ESBL/p(AmpC) *E. coli* and CTX^S^ *E. coli* isolates, revealed that most of them belong to commensal phylogroup A, followed by B1; 13 of them (11 CTX^S^ *E. coli* and 2 ESBL *E. coli* strains) being affiliated to pathogenic phylogroup B2. The dominance of phylogroup A in cows suffering from mastitis in Tunisia was reported by Saidani, et al. [20], and these findings agreed with previous studies that predominantly classified *E. coli* strains as commensal or opportunistic pathogens both in intramammary infections in dairy cattle or in food-producing animals [10,33,42]. Phylogenetic group analyses are an important approach to know the pathogenicity and the evolutionary relationships between different strains [43]. Indeed, identification of virulence factors revealed that strains defined as extra-intestinal pathogenic *E. coli* (ExPEC) and affiliated to pathogenic phylogroup B2 harbored two or three virulence genes (*stx2*, *cnf1*, *fimA*, and *aer*). The presence of cytotoxic necrotizing factor 1 (CNF1) and fimbriales adhesines type1 (*fimA*) led to classify *E. coli* isolates as ExPEC [10,44]. The absence or the low distribution of CNF genes *(cnf1/cnf2*) and *fimA* has been reported in bovine mastitis in dairy farms in Iran and Bangladesh [45,46]. In addition, the *aer* gene that encodes aerobactin receptor related to iron uptake was detected in our collection. Some studies reported a high prevalence of such gene in mastitis caused by *E. coli* in cattle [10,45].

It is important to highlight the presence of shigatoxin encoding gene (*stx2*) in our isolated strains. Several studies have reported shigatoxin genes (*stx1*/stx2), and shigatoxigenic *E. coli* (STEC) are considered the most pathogenic variants in bovine mastitis [8,9], although recent studies in Bangladsh and China have reported the absence of *stx* genes [46,47].

To our knowledge, this is the first description of STEC in clinical mastitis in dairy cattle in Tunisia. The effect of the virulence factors (VFs) of *E. coli* on the severity of dairy cattle suffering mastitis remains unclear. Thus, more research on different VFs of *E. coli* is necessary to better know the pathogenic relationship between bovine mammary infections and the severity of the clinical cases in Tunisia.

In the present study, MLST analysis was performed for ESBL/p(AmpC) *E. coli* and *K. pneumoniae* strains and revealed an extended diversity, with different STs being detected, especially in *E. coli* isolates (ST405, ST58, ST155, ST10, ST617, and ST399). Of note, none of our ESBL CTX-M-15 *E. coli* isolates belonged to ST131. Nevertheless, ST405 and ST155 were the most STs found in CTX-M-15 *E. coli* producers; the ST405 was considered as a vehicle driving CTX-M-15 worldwide and frequently associated with clinical human settings [32,48]. However, ST155 and ST405 were identified in healthy and sick chickens and cattle in Tunisia and Nigeria [11,19]. The ST58 and ST10 have been reported in *E. coli* isolates carrying CTX-M and IncFII plasmids, recovered from bovine mastitis, in France and Germany [27,28]; they were also highly distributed in livestock species in many African countries [11]. Furthermore, ST58 and ST10 have recently been related to *mcr-1* positive *E. coli* isolates from bovine mastitis in China [14]. Recently, ST58, ST155, and ST10 were identified in ESBL *E. coli* isolates from diarrheic poultry in Tunisia [19]. The Detection of ST617 CMY-2 *E. coli* isolates in our study was supported by its presence in CTX-M *E. coli* producer isolates from bovine mastitis in Tunisia [20] and from companion animals and domestic farms in Tanzania [11]. The four CTXM-15 *K. pneumoniae* strains isolated from different farms belonged to the ST471 and ST101; the ST471 not being a frequent clone detected in bovine mastitis in farms on the coast of Tunisia [19]. This could suggest that ST471 is particularly well adapted to a bovine host. Although ST101 has been reported in *K. pneumoniae* isolated from milk of cows suffering from mastitis [49], there are few reports describing the genetic characteristics of *K. pneumoniae* strains from healthy or mastitis diseased bovine.

## 4. Conclusions

It is important to highlight that bovine mastitis is caused by environmental pathogens such as *Enterobacteriaceae* strains harboring different antibiotic resistance genes. The emergence of CTXM-15 and CMY-2 plasmids associated to PMQR and integrons in cattle in Tunisia could complicate the treatment of bovine mastitis and present zoonotic potential risk of transmission to humans. The presence of multidrug-resistant bacteria, related to virulence factors, especially shigatoxin genes belonging to different sequence types, is alarming and indicates a potential risk of the circulation of virulent and resistant clones to humans, animals, and the environment through contaminated milk or milk products. The application of good hygiene practices throughout the dairy industry and the prudent use of antimicrobial agents against diseases affecting dairy cows are important issues that should be addressed at the global level. 

## 5. Methods

### 5.1. Strain Collection

The present study was carried out on a collection of *Enterobacteriaceae* isolates that recovered from 300 dairy cattle with clinical mastitis in Tunisia and identified in our previously published paper [23]. This collection was composed of 14 ESBL *Enterobacteriaceae* isolates (10 *E. coli* and 4 *K. pneumoniae*), 3 cefotaxime resistant *E. coli* isolates, and 50 cefotaxime susceptible *E. coli* isolates. All collected isolates were conserved in skim milk at −80 °C in our strain library in the laboratory and included in this study for further characterization.

### 5.2. Antimicrobial Susceptibility Testing and AmpC Confirmation

Antimicrobial susceptibility testing was conducted on Mueller-Hinton agar (Biolife, Milano, Italy) plates using the agar disk diffusion method, according to Clinical and Laboratory Standards Institute (CLSI) criteria [50]. The tested antibiotics were (µg/disc): ampicillin (10), ticarcillin (75), amoxicillin/clavulanic acid (20 + 10), cefoxitin (30), Ceftazidime (30), cefotaxime (30), Imipenem (10), ertapenem (10), gentamicin (10), tobramycin (10), streptomycin (10), nalidixic acid (30), ciprofloxacin (5), sulphonamides (200), trimethoprim/sulfamethoxazole (1.25 + 23.75), tetracycline (30), and chloramphenicol (30). *E. coli* strain ATCC 25922 was used as a control strain. A screening test for the detection of (AmpC) was carried out by the (DDST), according to the CLSI criteria [50].

### 5.3. Genomic Extraction, Polymerase Chain Reaction and Sequencing

The genomic DNA of all *Enterobacteriaceae* isolates was obtained using the boiling extraction method. The concentration and the purity of the extracted DNA were measured by a Nanodrop Spectrophotometer (Thermo scientific, NANODROP ONE, Waltham, Massachusetts, Etats-Unis). The detection of the different specific genes among *Enterobacteriaceae* isolates was performed by conventional PCR, in thermal cycler BIO-RAD T100. The reaction occurred in a 25 µL reaction mix consisting of 5 µL of 10× Dream DNA polymerase buffer, 0.05 mM dNTPs (Thermo Scientific, France), 1U of Dream Taq DNA polymerase (Thermo Scientific, HTDS, Rue du Saule Trapu, 91300 Massy, France), 0.5 µM of each primer (Carthagenomics Advanced Technologies, Rue des Métiers, Tunis, Tunisia), and 5 µL of template DNA. The amplification program differs according to the genes sought and are presented in the relative references of each group of genes in the following paragraphs. For the visualization of PCR products, 5 µL were subjected to 1 or 2% agarose gel electrophoresis containing ethidium bromide (0.5 mg/mL). Lengths of the amplicons were determined in comparison with a 100 bp or 1 kb ladder.

The sequencing of PCR products was performed by Sanger Sequencing.

### 5.4. Characterization of β-Lactamase Genes and Their Genetic Environment

The genes encoding TEM, SHV, OXA-1, CTX-M, and CMY type beta-lactamases were analyzed by PCR, followed by sequencing all ESBL-p(AmpC) positive isolates [19]. Nucleotides and their deduced amino acid sequences were compared with those stored in the GenBank database to confirm the specific type of beta-lactamase genes [51]. The IS*Ecp1*, IS*26*, and orf477 sequences, surrounding *bla*_CTX-M_ and *bla*_CMY_ genes, were analyzed by PCR, using previously described primers and conditions [31]. In addition, the genes of TEM, SHV, and OXA1 beta-lactamases were performed for all CTX^S^ *E. coli.*

### 5.5. Characterization of Integrons and Resistance Mechanisms to Non-b-Lactam Antibiotic

The presence of the *int*I1 and *int*I2 genes (encoding class 1 and class 2 integrases, respectively) and the 3′-conserved segment (3′-CS) (*qac*EΔ1-*sul*1 genes) of class 1 integrons, in the trimethoprim-sulphamethoxazole resistant isolates, were examined by PCR [39]. Variable regions of class 1 integrons were characterized by PCR and sequencing for all *int*I1-positive isolates [19]. The presence of genes associated with quinolone resistance (*qnrA, qnrB*, *qep*A, and *aac*(*6′*)-Ib), streptomycin (*str*A and *str*B), sulphonamides (*sul*1, *sul*2, and *sul*3), and tetracycline (*tet*A and *tet*B), was determined by PCR. The *aac(6′)-Ib* amplicons were sequenced to identify *aac(6′)-Ib-cr* variants [19,31].

### 5.6. Virulence Factors and Phylogeny Groups

Virulence factors and phylogeny groups of *E. coli* isolates were screened by single or multiplex PCR assays for the presence of the eight genes encoding the following virulence factors: *fimA* (encoding type 1 fimbriae), *papG* allele III (adhesin PapG class III), *cnf1* (cytotoxic necrotizing factor), *papC* (P fimbriae), *aer* (aerobactin iron uptake system) [encoding virulence factors often found in ExPEC isolates], *eae* (Intimin), and *bfp* (Type IV bundle forming pili) genes [19]. All isolates were screened for the serotypes O25a and O25b [52,53], as well as the new diffuse adhesion encoding *afa* operon (Gen Bank accession number FM955495), specific for isolates of O25b:H4 ST131 producing CTXM-15 [53]. In addition, *E. coli* strains were assigned to the phylogenetic groups A, B1, B2, or D, using a PCR strategy with specific primers for *chuA*, *yjaA*, and *TspE4.C2* determinants as previously described [43].

### 5.7. Molecular Typing of ESBL-p(AmpC) Enterobacteriaceae Isolates

The ESBL/p(AmpC) *E. coli* and *K. pneumoniae* isolates were characterized by multilocus-sequence typing (MLST), using PCR amplification of the standard seven housekeeping loci [19]. All the amplicons were sequenced and compared with the sequences deposited in MLST database (https://enterobase.warwick.ac.uk/species/ecoli/allele_st_search, 1 May 2022) and (http://bigsdb.pasteur.fr/klebsiella/primers_used.html, 15 May 2022), specific for *E. coli* and *K. pneumoniae*, respectively.

## Figures and Tables

**Table 1:** Features of 17 extended spectrum β-lactmase and p(AmpC) *Enterobacteriaceae* isolates.

Strains	Farm	Genes Encoding Beta-Lactamases	*bla*CTX-M Genetic Environment	Phenotypes of Resistance to Non β-Lactams	Other Resistance Genes Detected Outside Integrons	Gene Cassette Arrays in Class 1 Integron	Phylogroup and Virulence Genes	ST
*E. C 1*	1	*bla*CTX-M-15 + *bla*OXA-1	*ISEcp1-orf477*	TET NAL SUL SXT	*tetB, sul3, sul2*, *aac(6′)-Ib-cr*	*Int1, dfrA*17	A	405
*E. C 2*	5	*bla*CTX-M-15 + *bla*OXA-1	*ISEcp1-orf477*	TET SXT SUL	*tetB, sul2*	*Int1, dfrA17*	*B2, cnf1, stx2, aer*	405
*E. C 3*	8	*bla*CTX-M-15 + *bla*OXA-1 + *bla*TEM-1b	*ISEcp1-orf477*	TET NAL	*tetB, aac(6′)-Ib-cr*	−	*B2, aer, fimA, stx2*	58
*E. C 4*	2	*bla*CTX-M-15 + *bla*OXA-1 + *bla*TEM-1b	*IS26/ISEcp1-orf477*	TET SUL NAL SXT	*tetB, sul3, sul2, aac(6′)-Ib-cr*	*Int1, dfrA17*	*D, aer, stx2*	58
*E. C 5*	10	*bla*CTX-M-15	*ISEcp1-orf477*	TET STR	*tetB, strA*	−	B1	405
*E. C 6*	6	*bla*CTX-M-15	*ISEcp1-orf477*	TET	*tetB*	−	A	405
*E. C 7*	18	*bla*CTX-M-15	*IS26/ISEcp-orf477*	CIP NAL SUL	*qnrA, sul2*	−	A	155
*E. C 8*	20	*bla*CTX-M-15	*IS26/ISEcp1-orf477*	CIP NAL SUL	*qnrA, sul3*	−	A	10
*E. C 9*	23	*bla*CTX-M-15	*ISEcp1-orf477*	NAL SUL TET CHL STR SXT	*tetB, sul3, sul2, strA*	ND	A	155
*E. C 10*	9	*bla*CTX-M-15	*ISEcp1-orf477*	SUL TET	*tetB, strA*	−	A	58
E. C 11 *	11	*bla*CMY-2	*ISEcp1-orf477*	TET SULNAL SXT STR	*tetA, sul2*	*Int1, aadA1 + dfrA*1	B1	155
E. C 12 *	14	*bla*CMY-2	*ISEcp1-orf477*	TET SUL SXT	*tetA*	*Int1, dfrA17*	B1	399
E. C 13 *	16	*bla*CMY-2	*ISEcp1-orf477*	TET SUL NAL CIP	*tetB, qnrA, sul2*	−	B1	617
*Kp1*	1	*bla*CTX-M-15 + *bla*TEM-1b	*IS26/ISEcp1-orf477*	TET NAL SUL	*tet A, sul1, sul2, qnrB*	−	−	471
*Kp2*	2	*bla*CTX-M-15 + *bla*TEM-1b	*ISEcp1-orf477*	CIP NAL SUL SXT	*qnrB, sul2*	*Int1, dfrA*17	−	471
*Kp3*	3	*bla*CTX-M-15 + *bla*TEM-1b	*ISEcp1-orf477*	TET SUL SXT STR	*tetA, sul1, sul2*	*Int1, dfrA*17 + *aadA5*	−	101
*Kp4*	4	*bla*CTX-M-15 + *bla*TEM-1b	*IS26/ISEcp1-orf477*	TET NAL CIP	*tetA, aac(6′)-Ib-cr*	−	−	101

*EC*: *E. coli*; *Kp*: *K. pneumoniae*; NAL: nalidixic acid; CIP: ciprofloxacin; SUL: sulfonamide; SXT: trimethoprim/sulfamethoxazole; TET: tetracycline; CHL: chloramphenicol; STR: streptomycin; TOB: tobramycin; ST: sequence type; ND: integron not detected in trimethoprim/sulfamethoxazole resistance isolates; −: integron is not sought in strains susceptible to trimethoprim/sulfamethoxazole. *: *E. coli* p(AmpC) producing isolates.

**Table 2:** Phenotypic and genotypic characteristics of 50 cefotaxime susceptible *E. coli* isolates recovered from bovine mastitis milk.

Strains	Antibiotic Resistance Phenotypes	Resistance Genes	Class 1 Integron	Gene Cassette Arrays in Class 1 Integron	Phylogroup and Virulence Genes
***E. C 168***	TET AMP SXT TIC SUL FOX STR CHL	*TEM-1b, tetA, catA*	+	*dfrA12 + aadA2*	B1
***E. C 3***	TET AMP SUL NAL CIP TIC SXT	*TEM-1b, tetA, qnrA, sul2*	ND	−	B1
***E. C 78***	TET AMP SXT TIC SUL STR CHL	*TEM-1b, tetA, catA, strA*	+	*dfrA 1 + aadA1*	B2, *sxt2*, *cnf1*
***E. C 115***	TET AMP SXT TIC SUL STR	*TEM-1b, tetA*	+	*dfrA1 + aadA1*	B1
***E. C 81***	TET AMP SXT TIC SUL STR	*TEM-1b, tetA, strA, dfrA1^a^*	ND	−	B2, *fimA, stx2*
***E. C 82***	TET AMP SXT TIC SUL STR	*TEM-1b, tetA*	+	*dfrA12 + aadA2*	B2, *stx2*, *cnf1*
***E. C 85***	TET AMP SXT TIC SUL STR	*TEM-1b, tetB, strA*	ND	−	A
***E. C 91***	TET AMP SXT TIC SUL STR	*TEM-1b, tetA*	+	*dfrA12 + aadA2*	B1
***E. C 134***	TET AMP SXT TIC SUL STR	*TEM-1b*	+	*dfrA1 + aadA1*	B2, *aer, fimA, cnf1*
***E. C 162***	TET AMP SXT TIC SUL STR	*TEM-1b, tetB*	+	*dfrA1 + aadA1*	B2, *cnf1, stx2*
***E. C 164***	TET AMP SXT TIC SUL STR	*TEM-1b, tetA*	+	*dfrA1 + aadA1*	B2, *cnf1*
***E. C 167***	TET AMP SXT TIC SUL STR	*TEM-1b, tetA*	+	*dfrA1 + aadA1*	B1
***E. C 42***	TET AMP SXT TIC SUL STR	*TEM-1b, tetA, strA*	+	*dfrA 12 + aadA2*	B1
***E. C 184***	TET AMP SXT TIC SUL STR	*TEM-1b, tetA*	+	*dfrA17 + aadA5*	B1
***E. C 186***	TET AMP SXT TIC SUL STR	*TEM-1b, tetA*	+	*dfrA17 + aadA5*	B1
***E. C 196***	TET AMP SXT TIC SUL STR	*TEM-1b, tetB*	+	*dfrA17 + aadA5*	B1
***E. C 116***	TET SXT SUL NAL CIP STR	*tetA, qnrA*	+	*dfrA1 + aadA1*	B1
***E. C 123***	TET AMP CHL NAL CIP SXT	*TEM-1b, tetA, catA, qnrA, dfrA1 ^a^*	ND	−	B2, *cnf1*
***E. C 10***	TET AMP SXT TIC SUL	*TEM-1b, tetA, sul2, dfrA12 ^a^*	ND	−	B2, *aer, cnf1*
***E. C 28***	TET AMP SXT TIC SUL	*TEM-1b, tetA, sul2, dfrA1 ^a^*	ND	−	A
***E. C 29***	TET AMP SXT TIC SUL	*TEM-1b, tetA, dfrA1 ^a^*	ND	−	B1
***E. C 40***	TET AMP SXT TIC SUL	*TEM-1b, tetA, dfrA1 ^a^*	ND	−	B1
***E. C 171***	TET AMP SXT TIC SUL	*TEM-1b, tetA, dfrA1 ^a^*	ND	−	B2, *stx2, fimA*
***E. C 175***	TETAMP SXT TIC SUL	*TEM-1b, tetA, dfrA1 ^a^*	ND	−	B1
***E. C 30***	TET AMP SXT TIC SUL	*TEM-1b, tetA*	ND	−	B2, *stx2, aer, fimA*
***E. C 17***	AMP TIC NAL CIP	*TEM-1b, qnrA*	−	−	A
***E. C 18***	AMP TIC NAL CIP	*TEM-1b, qnrA*	−	−	A
***E. C 195***	AMP TIC TET STR	*TEM-1b, tetB, strA*	−	−	A
***E. C 137***	AMP SUL STR	*TEM-1b, sul2, strA*	−	−	A
***E. C 14***	TET AMP TIC	*TEM-1b, tetA*	−	−	A
***E. C 25***	TET AMP TIC	*TEM-1b, tetA*	−	−	B1
***E. C 39***	TET AMP TIC	*TEM-1b, tetB*	−	−	B1
***E. C 70***	TET AMP TIC	*TEM-1b, tetB*	−	−	B1
***E. C 31***	TET AMP TIC	*TEM-1b, tetB*	−	−	B2, *stx2, aer, cnf1*
***E. C 32***	TET AMP TIC	*TEM-1b, tetB*	−	−	A
***E. C 135***	TET SUL STR	*tetB, sul2, strA*			B1
***E. C 183***	FOX AMP	*TEM-1b*	−	−	A
***E. C 23***	AMP TIC	*TEM-1b*	−	−	A
***E. C 179***	AMP STR	*TEM-1b, strA*	−	−	A
***E. C 190***	TET STR	*tetB, strA*			A
***E. C 119***	AMP	*TEM-1b*	−	−	A
***E. C 146***	FOX	−	−	−	A
***E. C 147***	FOX	−	−	−	B1
***E. C 163***	AMP	*TEM-1b*	−	−	A
E. C 194 *(*n* = 6)	TET	*tetB*	−	−	A

EC: *E. coli*; NAL: AMP: ampicillin; TIC: ticarcillin; FOX: cefoxitin; NAL: nalidixic acid; CIP: ciprofloxacin; SUL: sulfonamide; SXT: trimethoprim/sulfamethoxazole; TET: tetracycline; CHL: chloramphenicol; STR: streptomycin; TOB: tobramycin. ND: Integron not detected in trimethoprim/sulfamethoxazole resistance isolates; −: integron is not sought in strains susceptible to trimethoprim/sulfamethoxazole; ^a^ gene detected outside of intergron; * same phenotype and genotype in six strains; +: Integron detected in trimethoprim/sulfamethoxazole resistance isolates.

## Data Availability

All supporting data, code, and protocols have been provided within the article or through Supplementary data files.

## Supplementary Materials

The following supporting information can be downloaded at: https://www.mdpi.com/article/10.3390/pathogens11080948/s1, Figure S1: Antimicrobial resistance rate of 50 E. coli cefotaxime susceptible analyzed in this study; Supplementary file: Fasta form of sequence of ESBL and β-lactamases genes, gene casstte of integron and sequence type genes generated in this study.
