# Supplementary material for: Lineages, Virulence Gene Associated and Integrons among Extended Spectrum β-Lactamase (ESBL) and CMY-2 Producing Enterobacteriaceae from Bovine Mastitis, in Tunisia
Source: Pathogens. 2022 Aug 22;11(8):948. doi: 10.3390/pathogens11080948 (PMC9416333; doi:10.3390/pathogens11080948)
Supplement: Supplementary file 1 [file pathogens-11-00948-s001.zip › pathogens-1770468-supplementary figure s1.pdf]

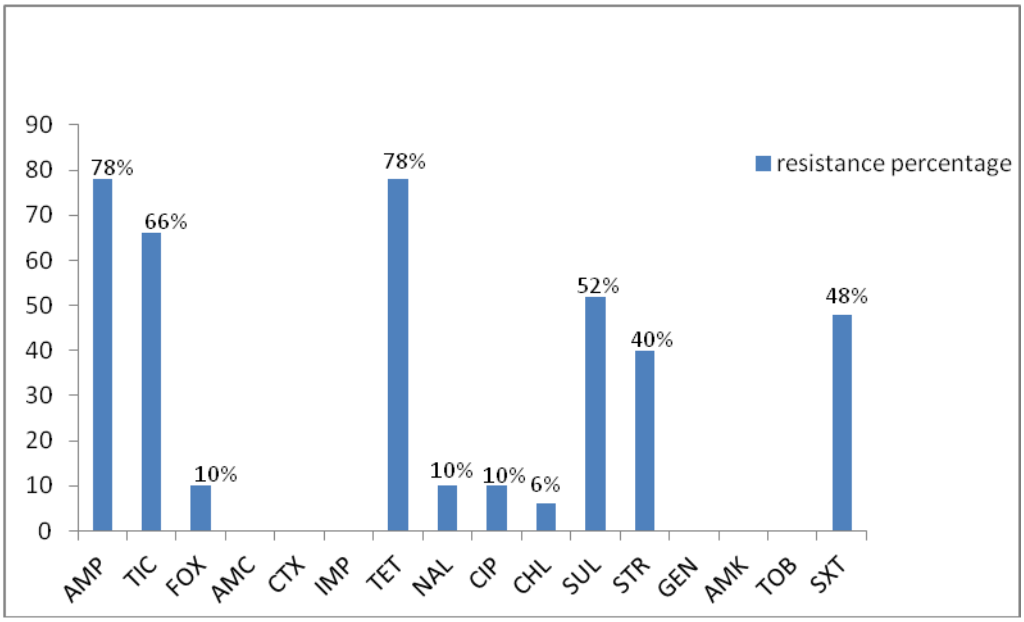

Figure S1. Antimicrobial resistance rate of 50 *E. coli* cefotaxime susceptible analyzed in this study. AMP: ampicillin; AMC: amoxicillin clavulanic acid; TIC: Ticarcillin, FOX: cefoxitin; CTX: céfotaxime ; CAZ: céftazidime; TET: tétracycline; NAL: nalidixic acid; CIP: ciprofloxacin; Sul: sulfonamide; SXT: trimethoprim-sulfamethoxazole; STR: streptomycin; CHL: Chloramphenicol, ;GEN: gentamicin; IMP: Imipenem; TOB: Tobramycin; % pourcentage.
