# Supplementary material for: Lineages, Virulence Gene Associated and Integrons among Extended Spectrum β-Lactamase (ESBL) and CMY-2 Producing Enterobacteriaceae from Bovine Mastitis, in Tunisia
Source: Pathogens. 2022 Aug 22;11(8):948. doi: 10.3390/pathogens11080948 (PMC9416333; doi:10.3390/pathogens11080948)
Supplement: Supplementary file 1 [file pathogens-11-00948-s001.zip › pathogens-1770468-supplementary file.pdf]

***CTXM-15* (757pb)**

KU163311.1 Escherichia coli strain 358B extended-spectrum beta-lactamase CTX-M-15 (blaCTX-M-15) gene, partial cds

>GCCAGTTCACGCTGATGGCGACGGCCACCGTCACGCTGTTGTTAGGAAGTGTGCCGCTGTATGCGCAAACGGCG
GACGTACAGCAAAAACTTGCCGAATTAGAGCGGCAGTCGGGAGGCAGACTGGGTGTGGCATTGATTAACACAGCA
GATAATTCGCAAATACTTTATCGTGCTGATGAGCGCTTTGCGATGTGCAGCACCAGTAAAGTGATGGCCGGGCCG
CGGTGCTGAAGAAAAGTGAAAGCGAACCGAATCTGTTAAATCAGCGAGTTGAGATCAAAAAATCTGACCTTGTTA
ACTATAATCCGATTGCGGAAAAGCACGTCAATGGGACGATGTCACTGGCTGAGCTTAGCGCGGCCGCGCTACAGT
ACAGCGATAACGTGGCGATGAATAAGCTGATTGCTCACGTTGGCGGCCCGGCTAGCGTCACCGCGTTCGCCCGAC
AGCTGGGAGACGAAACGTTCCGTCTCGACCGTACCGAGCCGACGTTAAACACCGCCATTCCGGGCGATCCGCGTG
ATACCACTTCACCTCGGGCAATGGCGCAAACTCTGCGGAATCTGACGCTGGGTAAAGCATTGGGCGACAGCCAAC
GGGCGCAGCTGGTGACATGGATGAAAGGCAATACCACCGGTGCAGCGAGCATTCAGGCTGGACTGCCTGCTTCCT
GGGTTGTGGGGGATAAAACCGGCAGCGGTGGCTATGGCACCACCAACGATATCGCGGTGATCTGGCCAAAAGATC
GTGCGCCG

***CTXM-15* (652pb)**

KU163311.1 Escherichia coli strain 358B extended-spectrum beta-lactamase CTX-M-15 (blaCTX-M-15) gene, partial cds

>GCCAGTTCACGCTGATGGCGACGGCCACCGTCACGCTGTTGTTAGGAAGTGTGCCGCTGTATGCGCAAACGGCG
GACGTACAGCAAAAACTTGCCGAATTAGAGCGGCAGTCGGGAGGCAGACTGGGTGTGGCATTGATTAACACAGCA
GATAATTCGCAAATACTTTATCGTGCTGATGAGCGCTTTGCGATGTGCAGCACCAGTAAAGTGATGGCCGGGCCG
CGGTGCTGAAGAAAAGTGAAAGCGAACCGAATCTGTTAAATCAGCGAGTTGAGATCAAAAAATCTGACCTTGTTA
ACTATAATCCGATTGCGGAAAAGCACGTCAATGGGACGATGTCACTGGCTGAGCTTAGCGCGGCCGCGCTACAGT
ACAGCGATAACGTGGCGATGAATAAGCTGATTGCTCACGTTGGCGGCCCGGCTAGCGTCACCGCGTTCGCCCGAC
AGCTGGGAGACGAAACGTTCCGTCTCGACCGTACCGAGCCGACGTTAAACACCGCCATTCCGGGCGATCCGCGTG
ATACCACTTCACCTCGGGCAATGGCGCAAACTCTGCGGAATCTGACGCTGGGTAAAGCATTGGGCGACAGCCAAC
GGGCGCAGCTGGTGACATGGATGAAAGGCAATACCACCGGTGCAGCGAGCATT

***CTXM-15* (757pb)**

KU163311.1 Escherichia coli strain 358B extended-spectrum beta-lactamase CTX-M-15 (blaCTX-M-15) gene, partial cds

>GCCAGTTCACGCTGATGGCGACGGCCACCGTCACGCTGTTGTTAGGAAGTGTGCCGCTGTATGCGCAAACGGCG
GACGTACAGCAAAAACTTGCCGAATTAGAGCGGCAGTCGGGAGGCAGACTGGGTGTGGCATTGATTAACACAGCA
GATAATTCGCAAATACTTTATCGTGCTGATGAGCGCTTTGCGATGTGCAGCACCAGTAAAGTGATGGCCGGGCCG
CGGTGCTGAAGAAAAGTGAAAGCGAACCGAATCTGTTAAATCAGCGAGTTGAGATCAAAAAATCTGACCTTGTTA
ACTATAATCCGATTGCGGAAAAGCACGTCAATGGGACGATGTCACTGGCTGAGCTTAGCGCGGCCGCGCTACAGT
ACAGCGATAACGTGGCGATGAATAAGCTGATTGCTCACGTTGGCGGCCCGGCTAGCGTCACCGCGTTCGCCCGAC
AGCTGGGAGACGAAACGTTCCGTCTCGACCGTACCGAGCCGACGTTAAACACCGCCATTCCGGGCGATCCGCGTG
ATACCACTTCACCTCGGGCAATGGCGCAAACTCTGCGGAATCTGACGCTGGGTAAAGCATTGGGCGACAGCCAAC
GGGCGCAGCTGGTGACATGGATGAAAGGCAATACCACCGGTGCAGCGAGCATTCAGGCTGGACTGCCTGCTTCCT
GGGTTGTGGGGGATAAAACCGGCAGCGGTGGCTATGGCACCACCAACGATATCGCGGTGATCTGGCCAAAAGATC
GTGCGCCG

***CTXM-15* (757pb)**

KU163311.1 Escherichia coli strain 358B extended-spectrum beta-lactamase CTX-M-15 (blaCTX-M-15) gene, partial cds

>GCCAGTTCACGCTGATGGCGACGGCCACCGTCACGCTGTTGTTAGGAAGTGTGCCGCTGTATGCGCAAACGGCG
GACGTACAGCAAAAACTTGCCGAATTAGAGCGGCAGTCGGGAGGCAGACTGGGTGTGGCATTGATTAACACAGCA
GATAATTCGCAAATACTTTATCGTGCTGATGAGCGCTTTGCGATGTGCAGCACCAGTAAAGTGATGGCCGGGCCG
CGGTGCTGAAGAAAAGTGAAAGCGAACCGAATCTGTTAAATCAGCGAGTTGAGATCAAAAAATCTGACCTTGTTA
ACTATAATCCGATTGCGGAAAAGCACGTCAATGGGACGATGTCACTGGCTGAGCTTAGCGCGGCCGCGCTACAGT
ACAGCGATAACGTGGCGATGAATAAGCTGATTGCTCACGTTGGCGGCCCGGCTAGCGTCACCGCGTTCGCCCGAC
AGCTGGGAGACGAAACGTTCCGTCTCGACCGTACCGAGCCGACGTTAAACACCGCCATTCCGGGCGATCCGCGTG
ATACCACTTCACCTCGGGCAATGGCGCAAACTCTGCGGAATCTGACGCTGGGTAAAGCATTGGGCGACAGCCAAC
GGGCGCAGCTGGTGACATGGATGAAAGGCAATACCACCGGTGCAGCGAGCATTCAGGCTGGACTGCCTGCTTCCT
GGGTTGTGGGGGATAAAACCGGCAGCGGTGGCTATGGCACCACCAACGATATCGCGGTGATCTGGCCAAAAGATC
GTGCGCCG

***CTXM-15* (757pb)**

KU163311.1 Escherichia coli strain 358B extended-spectrum beta-lactamase CTX-M-15 (blaCTX-M-15) gene, partial cds

>GCCAGTTCACGCTGATGGCGACGGCCACCGTCACGCTGTTGTTAGGAAGTGTGCCGCTGTATGCGCAAACGGCG
GACGTACAGCAAAAACTTGCCGAATTAGAGCGGCAGTCGGGAGGCAGACTGGGTGTGGCATTGATTAACACAGCA
GATAATTCGCAAATACTTTATCGTGCTGATGAGCGCTTTGCGATGTGCAGCACCAGTAAAGTGATGGCCGGGCCG
CGGTGCTGAAGAAAAGTGAAAGCGAACCGAATCTGTTAAATCAGCGAGTTGAGATCAAAAAATCTGACCTTGTTA
ACTATAATCCGATTGCGGAAAAGCACGTCAATGGGACGATGTCACTGGCTGAGCTTAGCGCGGCCGCGCTACAGT

ACAGCGATAACGTGGCGATGAATAAGCTGATTGCTCACGTTGGCGGCCCGGCTAGCGTCACCGCGTTCGCCCGAC
AGCTGGGAGACGAAACGTTCCGTCTCGACCGTACCGAGCCGACGTTAAACACCGCCATTCCGGGCGATCCGCGTG
ATACCACTTCACCTCGGGCAATGGCGCAAACTCTGCGGAATCTGACGCTGGGTAAAGCATTGGGCGACAGCCAAC
GGGCGCAGCTGGTGACATGGATGAAAGGCAATACCACCGGTGCAGCGAGCATTCAGGCTGGACTGCCTGCTTCCT
GGGTTGTGGGGGATAAAACCGGCAGCGGTGGCTATGGCACCACCAACGATATCGCGGTGATCTGGCCAAAAGATC
GTGCGCCG

CTXM-15 **(757pb)**

KU163311.1 Escherichia coli strain 358B extended-spectrum beta-lactamase CTX-M-15 (blaCTX-M-15) gene, partial cds

>GCCAGTTCACGCTGATGGCGACGGCCACCGTCACGCTGTTGTTAGGAAGTGTGCCGCTGTATGCGCAAACGGCG
GACGTACAGCAAAAACTTGCCGAATTAGAGCGGCAGTCGGGAGGCAGACTGGGTGTGGCATTGATTAACACAGCA
GATAATTCGCAAATACTTTATCGTGCTGATGAGCGCTTTGCGATGTGCAGCACCAGTAAAGTGATGGCCGGGCCG
CGGTGCTGAAGAAAAGTGAAAGCGAACCGAATCTGTTAAATCAGCGAGTTGAGATCAAAAAATCTGACCTTGTTA
ACTATAATCCGATTGCGGAAAAGCACGTCAATGGGACGATGTCACTGGCTGAGCTTAGCGCGGCCGCGCTACAGT
ACAGCGATAACGTGGCGATGAATAAGCTGATTGCTCACGTTGGCGGCCCGGCTAGCGTCACCGCGTTCGCCCGAC
AGCTGGGAGACGAAACGTTCCGTCTCGACCGTACCGAGCCGACGTTAAACACCGCCATTCCGGGCGATCCGCGTG
ATACCACTTCACCTCGGGCAATGGCGCAAACTCTGCGGAATCTGACGCTGGGTAAAGCATTGGGCGACAGCCAAC
GGGCGCAGCTGGTGACATGGATGAAAGGCAATACCACCGGTGCAGCGAGCATTCAGGCTGGACTGCCTGCTTCCT
GGGTTGTGGGGGATAAAACCGGCAGCGGTGGCTATGGCACCACCAACGATATCGCGGTGATCTGGCCAAAAGATC
GTGCGCCG

CTXM-15 **(757pb)**

KU163311.1 Escherichia coli strain 358B extended-spectrum beta-lactamase CTX-M-15 (blaCTX-M-15) gene, partial cds

>GCCAGTTCACGCTGATGGCGACGGCCACCGTCACGCTGTTGTTAGGAAGTGTGCCGCTGTATGCGCAAACGGCG
GACGTACAGCAAAAACTTGCCGAATTAGAGCGGCAGTCGGGAGGCAGACTGGGTGTGGCATTGATTAACACAGCA
GATAATTCGCAAATACTTTATCGTGCTGATGAGCGCTTTGCGATGTGCAGCACCAGTAAAGTGATGGCCGGGCCG
CGGTGCTGAAGAAAAGTGAAAGCGAACCGAATCTGTTAAATCAGCGAGTTGAGATCAAAAAATCTGACCTTGTTA
ACTATAATCCGATTGCGGAAAAGCACGTCAATGGGACGATGTCACTGGCTGAGCTTAGCGCGGCCGCGCTACAGT
ACAGCGATAACGTGGCGATGAATAAGCTGATTGCTCACGTTGGCGGCCCGGCTAGCGTCACCGCGTTCGCCCGAC
AGCTGGGAGACGAAACGTTCCGTCTCGACCGTACCGAGCCGACGTTAAACACCGCCATTCCGGGCGATCCGCGTG
ATACCACTTCACCTCGGGCAATGGCGCAAACTCTGCGGAATCTGACGCTGGGTAAAGCATTGGGCGACAGCCAAC
GGGCGCAGCTGGTGACATGGATGAAAGGCAATACCACCGGTGCAGCGAGCATTCAGGCTGGACTGCCTGCTTCCT
GGGTTGTGGGGGATAAAACCGGCAGCGGTGGCTATGGCACCACCAACGATATCGCGGTGATCTGGCCAAAAGATC
GTGCGCCG

CMY2 (1146pb)

ON412783.1 Escherichia coli IS1380-like element ISEc9 family transposase gene, partial cds; and class C beta-lactamase CMY-2 gene, complete cds

>ATGATGAAAAAATCGTTATGCTGCGCTCTGCTGCTGACAGCCTCTTTCTCCACATTTGCTGCCGCAAAAACAGA
ACAACAGATTGCCGATATCGTTAATCGCACCATCACCCCGTTGATGCAGGAGCAGGCTATTCCGGGTATGGCCGT
TGCCGTTATCTACCAGGGAAAACCCTATTATTTCACCTGGGGTAAAGCCGATATCGCCAATAACCACCCAGTCAC
GCAGCAAACGCTGTTTGAGCTAGGATCGGTTAGTAAGACGTTTAACGGCGTGTTGGGCGGCGATGCTATCGCCCG
CGGCGAAATTAAGCTCAGCGATCCGGTCACGAAATACTGGCCAGAACTGACAGGCAAACAGTGGCAGGGTATCCG
CCTGCTGCACTTAGCCACCTATACGGCAGGCGGCCTACCGCTGCAGATCCCCGATGACGTTAGGGATAAAGCCGC
ATTACTGCATTTTTATCAAAACTGGCAGCCGCAATGGACTCCGGGCGCTAAGCGACTTTACGCTAACTCCAGCAT
TGGTCTGTTTGGCGCGCTGGCGGTGAAACCCTCAGGAATGAGTTACGAAGAGGCAATGACCAGACGCGTCCTGCA
ACCATTAAAACTGGCGCATACCTGGATTACGGTTCCGCAGAACGAACAAAAAGATTATGCCTGGGGCTATCGCGA
AGGGAAGCCCGTACACGTTTCTCCGGGACAACTTGACGCCGAAGCCTATGGCGTGAAATCCAGCGTTATTGATAT
GGCCCGCTGGGTTCAGGCCAACATGGATGCCAGCCACGTTCAGGAGAAAACGCTCCAGCAGGGCATTGCGCTTGC
GCAGTCTCGCTACTGGCGTATTGGCGATATGTACCAGGGATTAGGCTGGGAGATGCTGAACTGGCCGCTGAAAGC
TGATTCGATCATCAACGGCAGCGACAGCAAAGTGGCATTGGCAGCGCTTCCCGCCGTTGAGGTAAACCCGCCCGC
CCCCGCAGTGAAAGCCTCATGGGTGCATAAAACGGGCTCCACTGGTGGATTTGGCAGCTACGTAGCCTTCGTTCC
AGAAAAAAACCTTGGCATCGTGATGCTGGCAAACAAAAGCTATCCTAACCCTGTCCGTGTCGAGGCGGCCTGGCG
CATTCTTGAAAAGCTGCAATAA

CMY2 **(901pb)**

ON412783.1 Escherichia coli IS1380-like element ISEc9 family transposase gene, partial cds; and class C beta-lactamase CMY-2 gene, complete cds

>ATGATGAAAAAATCGTTATGCTGCGCTCTGCTGCTGACAGCCTCTTTCTCCACATTTGCTGCCGCAAAAACAGA
ACAACAGATTGCCGATATCGTTAATCGCACCATCACCCCGTTGATGCAGGAGCAGGCTATTCCGGGTATGGCCGT
TGCCGTTATCTACCAGGGAAAACCCTATTATTTCACCTGGGGTAAAGCCGATATCGCCAATAACCACCCAGTCAC
GCAGCAAACGCTGTTTGAGCTAGGATCGGTTAGTAAGACGTTTAACGGCGTGTTGGGCGGCGATGCTATCGCCCG
CGGCGAAATTAAGCTCAGCGATCCGGTCACGAAATACTGGCCAGAACTGACAGGCAAACAGTGGCAGGGTATCCG
CCTGCTGCACTTAGCCACCTATACGGCAGGCGGCCTACCGCTGCAGATCCCCGATGACGTTAGGGATAAAGCCGC
ATTACTGCATTTTTATCAAAACTGGCAGCCGCAATGGACTCCGGGCGCTAAGCGACTTTACGCTAACTCCAGCAT

TGGTCTGTTTGGCGCGCTGGCGGTGAAACCCTCAGGAATGAGTTACGAAGAGGCAATGACCAGACGCGTCCTGCA
ACCATTAAAACTGGCGCATACCTGGATTACGGTTCCGCAGAACGAACAAAAAGATTATGCCTGGGGCTATCGCGA
AGGGAAGCCCGTACACGTTTCTCCGGGACAACTTGACGCCGAAGCCTATGGCGTGAAATCCAGCGTTATTGATAT
GGCCCGCTGGGTTCAGGCCAACATGGATGCCAGCCACGTTCAGGAGAAAACGCTCCAGCAGGGCATTGCGCTTGC
GCAGTCTCGCTACTGGCGTATTGGCGATATGTACCAGGGATTAGGCTGGGAGATGCTGAACTGGCCGCTGAAAGC
TG

CMY2 **(1040pb)**

ON412783.1 Escherichia coli IS1380-like element ISEc9 family transposase gene, partial cds; and class C beta-lactamase CMY-2 gene, complete cds

>ATGATGAAAAAATCGTTATGCTGCGCTCTGCTGCTGACAGCCTCTTTCTCCACATTTGCTGCCGCAAAAACAGA
ACAACAGATTGCCGATATCGTTAATCGCACCATCACCCCGTTGATGCAGGAGCAGGCTATTCCGGGTATGGCCGT
TGCCGTTATCTACCAGGGAAAACCCTATTATTTCACCTGGGGTAAAGCCGATATCGCCAATAACCACCCAGTCAC
GCAGCAAACGCTGTTTGAGCTAGGATCGGTTAGTAAGACGTTTAACGGCGTGTTGGGCGGCGATGCTATCGCCCG
CGGCGAAATTAAGCTCAGCGATCCGGTCACGAAATACTGGCCAGAACTGACAGGCAAACAGTGGCAGGGTATCCG
CCTGCTGCACTTAGCCACCTATACGGCAGGCGGCCTACCGCTGCAGATCCCCGATGACGTTAGGGATAAAGCCGC
ATTACTGCATTTTTATCAAAACTGGCAGCCGCAATGGACTCCGGGCGCTAAGCGACTTTACGCTAACTCCAGCAT
TGGTCTGTTTGGCGCGCTGGCGGTGAAACCCTCAGGAATGAGTTACGAAGAGGCAATGACCAGACGCGTCCTGCA
ACCATTAAAACTGGCGCATACCTGGATTACGGTTCCGCAGAACGAACAAAAAGATTATGCCTGGGGCTATCGCGA
AGGGAAGCCCGTACACGTTTCTCCGGGACAACTTGACGCCGAAGCCTATGGCGTGAAATCCAGCGTTATTGATAT
GGCCCGCTGGGTTCAGGCCAACATGGATGCCAGCCACGTTCAGGAGAAAACGCTCCAGCAGGGCATTGCGCTTGC
GCAGTCTCGCTACTGGCGTATTGGCGATATGTACCAGGGATTAGGCTGGGAGATGCTGAACTGGCCGCTGAAAGC
TGATTCGATCATCAACGGCAGCGACAGCAAAGTGGCATTGGCAGCGCTTCCCGCCGTTGAGGTAAACCCGCCCGC
CCCCGCAGTGAAAGCCTCATGGGTGCATAAAACGGGCTCCACTGGTGGATTTGGCAGCTACGTAGC

OXA-1 **(1031pb)**

MH208296.1 Escherichia coli strain Sy16 class 1 integron oxacillin-hydrolyzing class D beta-lactamase OXA-1 (blaOXA) gene, blaOXA-1 allele and aminoglycoside 3'- (9) - O - adenyltransferase (aadA1) gene, complete cds

>ACGCAGCAGGGCAGTCGCCCTAAAACAAAGTTGGGCGAACCCGGAGCCTCATTAATTGTTAGCCGTTAAAATTA
AGCCCTTTACCAAACCAATACTTATTATGAAAAACACAATACATATCAACTTCGCTATTTTTTTAATAATTGCAA
ATATTATCTACAGCAGCGCCAGTGCATCAACAGATATCTCTACTGTTGCATCTCCATTATTTGAAGGAACTGAAG
GTTGTTTTTTACTTTACGATGCATCCACAAACGCTGAAATTGCTCAATTCAATAAAGCAAAGTGTGCAACGCAAA
TGGCACCAGATTCAACTTTCAAGATCGCATTATCACTTATGGCATTTGATGCGGAAATAATAGATCAGAAAACCA
TATTCAAATGGGATAAAACCCCCAAAGGAATGGAGATCTGGAACAGCAATCATACACCAAAGACGTGGATGCAAT
TTTCTGTTGTTTGGGTTTCGCAAGAAATAACCCAAAAAATTGGATTAAATAAAATCAAGAATTATCTCAAAGATT
TTGATTATGGAAATCAAGACTTCTCTGGAGATAAAGAAAGAAACAACGGATTAACAGAAGCATGGCTCGAAAGTA
GCTTAAAAATTTCACCAGAAGAACAAATTCAATTCCTGCGTAAAATTATTAATCACAATCTCCCAGTTAAAAACT
CAGCCATAGAAAACACCATAGAGAACATGTATCTACAAGATCTGGATAATAGTACAAAACTGTATGGGAAAACTG
GTGCAGGATTCACAGCAAATAGAACCTTACAAAACGGATGGTTTGAAGGGTTTATTATAAGCAAATCAGGACATA
AATATGTTTTTGTGTCCGCACTTACAGGAAACTTGGGGTCGAATTTAACATCAAGCATAAAAGCCAAGAAAAATG
CGATCACCATTCTAAACACACTAAATTTATAAAAAATCTAATGGCAAAATCGCCCAACCCTTCAATCAAGTCGGG
ACGGCCAAAAGCAAGCTTTTGGCTCCCCTCGCTGGCGCTCGGCGCCCCTTATTTCAA

TEMC **(1069pb)**

CP099722.1 Escherichia coli strain EC21GDE043 plasmid pGDE043-mcr1, complete sequence

>TTCTTGAAGACGAAAGGGCCTCGTGATACGC**C**TATTTTTATAGGTTAATGTCATGATAATAATGGTTTCTTAGA
CGTCAGGTGGCACTTTTCGGGGAAATGTGCGCGGAACCCCTATTTGTTTATTTTTCTAAATACATT**C**AAATA**T**GT
ATCCGCTCATGA**G**ACAATAACCCTG**G**TAAATGCTTCAATAATATTGAAAAAGGAAGAGTATGAGTATTCAACATT
TTCGTGTCGCCCTTATTCCCTTTTTTGCGGCATTTTGC**C**TTCCTGTTTTTGCTCACCCAGAAACGCTGGTGAAAG
TAAAAGATGCTGAAGAT**C**AGTTGGGTGCACGAGTGGGTTACATCGAACTGGATCTCAACAGCGGTAAGATCCTTG
AGAGTTTTCGCCCCGAAGAACGTTTTCCAATGATGAGCACTTTTAAAGTTCTGCTATGTGGTGCGGTATTATCCC
GTGTTGACGCCGGGCAAGAGCAACTCGGTCGCCGCATACACTATTCTCAGAATGACTTGGTT**G**AGTACTCACCAG
TCACAGAAAAGCATCTTACGGATGGCATGACAGTAAGAGAATTATGCAGTGCTGCCATAACCATGAGTGATAACA
CTGC**T**GCCAACTTACTTCTGACAACGATCGGAGGACCGAAGGAGCTAACCGCTTTTTTGCACAACATGGGGGATC
ATGTAAC**T**CGCCTTGAT**CG**TTGGGAACCGGAGCTGAATGAAGCCATACCAAACGACGAGCGTGACACCACGATGC
CTGCAGCAATGGCAACAACGTTGCGCAAACTATTAACTGGCGAACTACTTACTCTAGCTTCCCGGCAACAATTAA
TAGACTGGATGGAGGCGGATAAAGTTGCAGGACCACTTCTGCGCTCGGCCCTTCCGGCTGGCTGGTTTATTGCTG
ATAAATCTGGA**G**CC**G**GT**G**AGCGTGG**G**TCTCGCGGTATCATTGCAGCACTGGGGCCAGATGGTAAGCCCTCCCGTA
TCGTAGTTATCTACA**C**GACGGGGAGTCAGGCAACTATGGATGAACGAAATAGACAGATCGCTGAGATAGGTGCCT
CACTGATTAAGCATTGGTAA

TEMC **(739pb)**

CP099722.1 Escherichia coli strain EC21GDE043 plasmid pGDE043-mcr1, complete sequence

>TTCTTGAAGACGAAAGGGCCTCGTGATACGC**C**TATTTTTATAGGTTAATGTCATGATAATAATGGTTTCTTAGA
CGTCAGGTGGCACTTTTCGGGGAAATGTGCGCGGAACCCCTATTTGTTTATTTTTCTAAATACATT**C**AAATA**T**GT

ATCCGCTCATGA**G**ACAATAACCCTG**G**TAAATGCTTCAATAATATTGAAAAAGGAAGAGTATGAGTATTCAACATT
T**T**CGTGTCGCCCTTATTCCCTTTTTTGCGGCATTTTGC**C**TTCCTGTTTTTGCTCACCCAGAAACGCTGGTGAAAG
TAAAAGATGCTGAAGAT**C**AGTTGGGTGCACGAGTGGGTTACATCGAACTGGATCTCAACAGCGGTAAGATCCTTG
AGAGTTTTCGCCCCGAAGAACGTTTTCCAATGATGAGCACTTTTAAAGTTCTGCTATGTGGTGCGGTATTATCCC
GTGTTGACGCCGGGCAAGAGCAACTCGGTCGCCGCATACACTATTCTCAGAATGACTTGGTT**G**AGTACTCACCAG
TCACAGAAAAGCATCTTACGGATGGCATGACAGTAAGAGAATTATGCAGTGCTGCCATAACCATGAGTGATAACA
CTGC**T**GCCAACTTACTTCTGACAACGATCGGAGGACCGAAGGAGCTAACCGCTTTTTTGCACAACATGGGGGATC
ATGTAAC**T**CGCCTTGAT**CG**TTGGGAACCGGAGCTGAATGAAGCCATACCAAACGACGAGCGTGAC

***TEMC* (974pb)**
CP099722.1 Escherichia coli strain EC21GDE043 plasmid pGDE043-mcr1, complete sequence
>TTCTTGAAGACGAAAGGGCCTCGTGATACGC**C**TATTTTTATAGGTTAATGTCATGATAATAATGGTTTCTTAGA
CGTCAGGTGGCACTTTTCGGGGAAATGTGCGCGGAACCCCTATTTGTTTATTTTTCTAAATACATT**C**AAATA**T**GT
ATCCGCTCATGA**G**ACAATAACCCTG**G**TAAATGCTTCAATAATATTGAAAAAGGAAGAGTATGAGTATTCAACATT
T**T**CGTGTCGCCCTTATTCCCTTTTTTGCGGCATTTTGC**C**TTCCTGTTTTTGCTCACCCAGAAACGCTGGTGAAAG
TAAAAGATGCTGAAGAT**C**AGTTGGGTGCACGAGTGGGTTACATCGAACTGGATCTCAACAGCGGTAAGATCCTTG
AGAGTTTTCGCCCCGAAGAACGTTTTCCAATGATGAGCACTTTTAAAGTTCTGCTATGTGGTGCGGTATTATCCC
GTGTTGACGCCGGGCAAGAGCAACTCGGTCGCCGCATACACTATTCTCAGAATGACTTGGTTGAGTACTCACCAG
TCACAGAAAAGCATCTTACGGATGGCATGACAGTAAGAGAATTATGCAGTGCTGCCATAACCATGAGTGATAACA
CTGC**T**GCCAACTTACTTCTGACAACGATCGGAGGACCGAAGGAGCTAACCGCTTTTTTGCACAACATGGGGGATC
ATGTAAC**T**CGCCTTGAT**CG**TTGGGAACCGGAGCTGAATGAAGCCATACCAAACGACGAGCGTGACACCACGATGC
CTGCAGCAATGGCAACAACGTTGCGCAAACTATTAACTGGCGAACTACTTACTCTAGCTTCCCGGCAACAATTAA
TAGACTGGATGGAGGCGGATAAAGTTGCAGGACCACTTCTGCGCTCGGCCCTTCCGGCTGGCTGGTTTATTGCTG
ATAAATCTGGA**G**CC**G**GT**G**AGCGTGG**G**TCTCGCGGTATCATTGCAGCACTGGGGCCAGATGGTAAGCCCTCCCGTA
***TEMC* (1049pb)**
CP099722.1 Escherichia coli strain EC21GDE043 plasmid pGDE043-mcr1, complete sequence
>TTCTTGAAGACGAAAGGGCCTCGTGATACGC**C**TATTTTTATAGGTTAATGTCATGATAATAATGGTTTCTTAGA
CGTCAGGTGGCACTTTTCGGGGAAATGTGCGCGGAACCCCTATTTGTTTATTTTTCTAAATACATT**C**AAATA**T**GT
ATCCGCTCATGA**G**ACAATAACCCTG**G**TAAATGCTTCAATAATATTGAAAAAGGAAGAGTATGAGTATTCAACATT
TTCGTGTCGCCCTTATTCCCTTTTTTGCGGCATTTTGC**C**TTCCTGTTTTTGCTCACCCAGAAACGCTGGTGAAAG
TAAAAGATGCTGAAGAT**C**AGTTGGGTGCACGAGTGGGTTACATCGAACTGGATCTCAACAGCGGTAAGATCCTTG
AGAGTTTTCGCCCCGAAGAACGTTTTCCAATGATGAGCACTTTTAAAGTTCTGCTATGTGGTGCGGTATTATCCC
GTGTTGACGCCGGGCAAGAGCAACTCGGTCGCCGCATACACTATTCTCAGAATGACTTGGTT**G**AGTACTCACCAG
TCACAGAAAAGCATCTTACGGATGGCATGACAGTAAGAGAATTATGCAGTGCTGCCATAACCATGAGTGATAACA
CTGCTGCCAACTTACTTCTGACAACGATCGGAGGACCGAAGGAGCTAACCGCTTTTTTGCACAACATGGGGGATC
ATGTAAC**T**CGCCTTGAT**CG**TTGGGAACCGGAGCTGAATGAAGCCATACCAAACGACGAGCGTGACACCACGATGC
CTGCAGCAATGGCAACAACGTTGCGCAAACTATTAACTGGCGAACTACTTACTCTAGCTTCCCGGCAACAATTAA
TAGACTGGATGGAGGCGGATAAAGTTGCAGGACCACTTCTGCGCTCGGCCCTTCCGGCTGGCTGGTTTATTGCTG
ATAAATCTGGA**G**CC**G**GT**G**AGCGTGG**G**TCTCGCGGTATCATTGCAGCACTGGGGCCAGATGGTAAGCCCTCCCGTA
TCGTAGTTATCTACA**C**GACGGGGAGTCAGGCAACTATGGATGAACGAAATAGACAGATCGCTGAGATAGGTGCCT
***aac-6'(Ib)-cr*: (755pb)**
NG_067971.1 Serratia marcescens subsp. marcescens S89 pPCMI3 aac(6')-Ib-cr gene for fluoroquinolone-acetylating aminoglycoside 6'-N-acetyltransferase AAC(6')-Ib-cr9, complete CDS
>GTTGCTTCGTGGCGGCGCTTGCGTGCTACGCTAAGCTTCGCACGCCGCTTGCCACTGCGCACCGCGGCTTAACT
CAGGCGTTAGCCACCAAGAAGGTGCCATGAAAACATTTAACGATTCCGTCACACTGCGCCTCATGACTGAGCATG
ACCTTGCGATGCTCTATGAGTGGCTAAATCGATCTCATATCGTCGAGTGGTGGGGCGGAGAAGAAGCACGCCCGA
CACTTGCTGACGTACAGGAACAGTACTTGCCAAGCGTTTTAGCGCAAGAGTCCGTCACTCCATACATTGCAATGC
TGAATGGAGAGCCGATTGGGTATGCCCAGTCGTACGTTGCTCTTGGAAGCGGGGACGGAAGGTGGGAAGAAGAAA
CCGATCCAGGAGTACGCGGAATAGACCAGTTACTGGCGAATGCATCACAACTGGGCAAAGGCTTGGGAACCAAGC
TGGTTCGAGCTCTGGTTGAGTTGCTGTTCAATGATCCCGAGGTCACCAAGATCCAAACGGACCCGTCGCCGAGCA
ACTTGCGAGCGATCCGATGCTACGAGAAAGCGGGGTTTGAGAGGCAAGGTACCGTAACCACCCCATATGGTCCAG
CCGTGTACATGGTTCAAACACGCCAGGCATTCGAGCGAACACGCAGTGATGCCTAACCCTTCCATCGAGGGGGAC
GTCCAAGGGCTGGCGCCCTTGGCCGCCCCTCATGTCAAACGTTGAGCGACTGCTTACCCGTTTGACGGTCGGCCG
CTCCTG
***aac-6'(Ib)-cr*: (654pb)**
NG_067971.1 Serratia marcescens subsp. marcescens S89 pPCMI3 aac(6')-Ib-cr gene for fluoroquinolone-acetylating aminoglycoside 6'-N-acetyltransferase AAC(6')-Ib-cr9, complete CDS
>GTTGCTTCGTGGCGGCGCTTGCGTGCTACGCTAAGCTTCGCACGCCGCTTGCCACTGCGCACCGCGGCTTAACT
CAGGCGTTAGCCACCAAGAAGGTGCCATGAAAACATTTAACGATTCCGTCACACTGCGCCTCATGACTGAGCATG
ACCTTGCGATGCTCTATGAGTGGCTAAATCGATCTCATATCGTCGAGTGGTGGGGCGGAGAAGAAGCACGCCCGA
CACTTGCTGACGTACAGGAACAGTACTTGCCAAGCGTTTTAGCGCAAGAGTCCGTCACTCCATACATTGCAATGC
TGAATGGAGAGCCGATTGGGTATGCCCAGTCGTACGTTGCTCTTGGAAGCGGGGACGGAAGGTGGGAAGAAGAAA
CCGATCCAGGAGTACGCGGAATAGACCAGTTACTGGCGAATGCATCACAACTGGGCAAAGGCTTGGGAACCAAGC

TGGTTCGAGCTCTGGTTGAGTTGCTGTTCAATGATCCCGAGGTCACCAAGATCCAAACGGACCCGTCGCCGAGCA
ACTTGCGAGCGATCCGATGCTACGAGAAAGCGGGGTTTGAGAGGCAAGGTACCGTAACCACCCCATATGGTCCAG
CCGTGTACATGGTTCAAACACGCCAGGCATTCGAGCGAACACGCAGTGATGCCTA

dfrA17 **(474pb)**

NG_047710.1 Escherichia coli GH326 dfrA17 gene for trimethoprim-resistant dihydrofolate reductase DfrA17, complete CDS

>TTGAAAATATCATTGATTTCTGCAGTGTCAGAAAATGGCGTAATCGGTAGTGGTCCTGATATCCCGTGGTCAGT
AAAAGGTGAGCAACTACTCTTTAAAGCGCTCACATATAATCAATGGCTCCTTGTCGGAAGAAAAACATTTGACTC
TATGGGTGTTCTTCCAAATCGCAAATATGCAGTAGTGTCAAAGAACGGAATTTCAAGCTCAAATGAAAACGTCCT
AGTTTTTCCTTCAATAGAAAATGCTTTGAAAGAGCTATCAAAAGTTACAGATCATGTATATGTCTCTGACGGGGG
TCAAATCTATAATAGCCTTATTGAAAAAGCAGATATAATTCATTTGTCTACTGTTCACGTTGAAGTCGAAGGTGA
TATCAAATTCCCTATAATGCCTGAGAATTTCAATTTGGTTTTTGAACAGTTTTTTATGTCTAATATAAATTATAC
ATACCAGATTTGGAAAAAAGGCTAA

Gene cassette of integron class1: *dfrA1+aadA1* (1112pb)

CP054237.1 Escherichia coli strain EcPF5 plasmid p1, complete sequence

>GAGTTATCGGGAATGGCCCTGATATTCCATGGAGTGCCAAAGGTGAACAGCTCCTGTTTAAAGCTATTACCTAT
AACCAATGGCTGTTGGTTGGACGCAAGACTTTTGAATCAATGGGAGCATTACCCAACCGAAAGTATGCGGTCGTA
ACACGTTCAAGTTTTACATCTGACAATGAGAACGTAGTGATCTTTCCATCAATTAAAGATGCTTTAACCAACCTA
AAGAAAATAACGGATCATGTCATTGTTTCAGGTGGTGGGGAGATATACAAAAGCCTGATCGATCAAGTAGATACA
CTACATATATCTACAATAGACATCGAGCCGGAAGGTGATGTTTACTTTCCTGAAATCCCCAGCAATTTTAGGCCA
GTTTTTACCCAAGACTTCGCCTCTAACATAAATTATAGTTACCAAATCTGGCAAAAGGGTTAACAAGTGGCAGCA
ACGGATTCGCAAACCTGTCACGCCTTTTGTACCAAAAGCCGCGCCAGGTTTGCGATCCGCTGTGCCAGGCGTTAA
ACATCATGAGGGAAGCGGTGATCGCCGAAGTATCGACTCAACTATCAGAGGTAGTTGGCGTCATCGAGCGCCATC
TCGAACCGACGTTGCTGGCCGTACATTTGTACGGCTCCGCAGTGGATGGCGGCCTGAAGCCACACAGTGATATTG
ATTTGCTGGTTACGGTGACCGTAAGGCTTGATGAAACAACGCGGCGAGCTTTGATCAACGACCTTTTGGAAACTT
CGGCTTCCCCTGGAGAGAGCGAGATTCTCCGCGCTGTAGAAGTCACCATTGTTGTGCACGACGACATCATTCCGT
GGCGTTATCCAGCTAAGCGCGAACTGCAATTTGGAGAATGGCAGCGCAATGACATTCTTGCAGGTATCTTCGAGC
CAGCCACGATCGACATTGATCTGGCTATCTTGCTGACAAAAGCAAGAGAACATAGCGTTGCCTTGGTAGGTCCAG
CGGCGGAGGAACTCTTTGATCCGGTTCCTGAACAGGATCTATTTGAGGCGCTAAATGAAACCTTAACGCTATGGA
ACTCGCCGCCCGACTGGGCTGGCGATGAGCGAAATGTAGTGCTTACGTTGTCCCGCATTTGG

Gene cassette of integron class1 *dfrA17+aadA1* (1120pb)

AM937244.2 Escherichia coli class I integron containing dhfr17 and aadA5 genes

>CGCGTTACGCCGTGGGTCGATGTTTGATGTTATGGAGCAGCAACGATGTTACGCAGCAGGGCAGTCGCCCTAAA
ACAAAGTTAGCCATTAAGGGAGTTAAATTGAAAATATCATTGATTTCTGCAGTGTCAGAAAATGGCGTAATCGGT
AGTGGTCCTGATATCCCGTGGTCAGTAAAAGGTGAGCAACTACTCTTTAAAGCGCTCACATATAATCAATGGCTC
CTTGTCGGAAGAAAAACATTTGACTCTATGGGTGTTCTTCCAAATCGCAAATATGCAGTAGTGTCAAAGAACGGA
ATTTCAAGCTCAAATGAAAACGTCCTAGTTTTTCCTTCAATAGAAAATGCTTTGAAAGAGCTATCAAAAGTTACA
GATCATGTATATGTCTCTGGCGGGGGTCAAATCTATAATAGCCTTATTGAAAAAGCAGATATAATTCATTTGTCT
ACTGTTCACGTTGAAGTCGAAGATGATATCAAATTCCCTATAATGCCTGAGAATTTCAATTTGGTTTTTGAACAG
TTTTTTATGTCTAATATAAATTATACATACCAGATTTGGAAAAAAGGCTAACAATGCGTTGCAGCACCAGTCGCT
TCGCTCCTTGGACAGCTTTTAAGTCGTGTCTTTGTGGTTTTGCTGCGCAAAAGTATTCCACAAAGCCGCAACTTA
AAAGCTGCCGCTGAACTTAACGTTAGGCATCATGGGTGAATTTTTCCCTGCACAAGTTTTCAAGCAGCTGTCCCA
CGCTCGCGCGGTGATCGAGCGCCATCTGGCTGCGACACTGGACACAATCCACCTGTTCGGATCTGCGATCGATGG
AGGGCTGAAGCCGGACAGCGACATAGACTTGCTCGTGACCGTCAGCGCCGCACCTAACGATTCGCTCCGGCAGGC
GCTAATGCTCGATTTGCTGAAAGTCTCATCACCGCCAGGCGATGGCGGAACATGGCGACCGCTGGAGCTAACTGT
TGTCGCTCGAAGCGAAGTAGTGCCTTGGCGCTATCCGGCGCGGCGTGAGCTTCAGTTCGGTGAGTGGCTCCGCCA
CGACATCCTTTCCGGAACGTTCGAGCCTGCCGTTCTGGATCACGATCTTGCGATTTTGCTGACCAAGGCGA

dfrA12+aadA2 (**980pb)**

DQ995286.1 Acinetobacter baumannii class I integron dihydrofolate reductase (dhrfXII) and streptomycin/spectinomycin 3' adenyltransferase (aadA2) genes, complete cds

>CGTACGCCGTGGGTCGATGTTTGATGTTATGGAGCAGCAACGATGTTACGCAGCAGGGCAGTCGCCCTAAAACA
AAGTTAGCCATATGAACTCGGAATCAGTACGCATTTATCTCGTTGCTGCGATGGGAGCCAATCGGGTTATTGGCA
ATGGTCCTAATATCCCCTGGAAAATTCCGGGTGAGCAGAAGATTTTTCGCAGACTCACTGAGGGAAAAGTCGTTG
TCATGGGGCGAAAGACCTTTGAGTCTATCGGCAAGCCTCTACCGAACCGTCACACATTGGTAATCTCACGCCAAG
CTAACTACCGCGCCACTGGCTGCGTAGTTGTTTCAACGCTGTCGCACGCTATCGCTTTGGCATCCGAACTCGGCA
ATGAACTCTACGTCGCGGGCGGAGCTGAGATATACACTCTGGCACTACCTCACGCCCACGGCGTGTTTCTATCTG
AGGTACATCAAACCTTCGAGGGTGACGCCTTCTTCCCAATGCTCAACGAAACAGAATTCGAGCTTGCCTCAACCG
AAACCATTCAAGCTGTAATTCCGTACACCCACTCCGTTTATGCGCGTCGAAACGGCTAACCATTCCGTCAACGGG
ACGCCAAAATGCTGCGCATTTTGGTTCCCTCCGCTGCGCTCCGGCTCTCGTTACGTCCAACGTTAGCACCACTGA
AACCCAGCTTTATTTAGCTCATGTTTATTCAAACGGCATTTAGCTTTTCAGGCGTTATTCAGTGCCTGTTTTGCC
TTTTTTCCGGGCTTCGCCTGCATGGGCTGCGCAGGTTTTCAGTCTTTTTGGCCTCTAGCCCTTGCGTAGCAAGCG

CAAGCAGCTATCGTTTTTGCAGTGCTGTGCCGCCTCGGTGGCGCAGCGTTTTTTCACGGTTAGCGCCCGTCGCCA
AATTCAAGTTATCCGTTTTGGCTTCTGGTTCTAACATTTCGGTCAAGCCGACCCGCATTCTGCGGTCGGCTTACC
TCGCCC

Sequences of 7 seven housekeeping genes of *E. coli*

ST 405

>adk35
GGGGAAAGGGACTCAGGCTCAGTTCATCATGGAGAAATATGGTATTCCGCAAATCTCCACTGGCGATATGCTGCG
TGCTGCGGTCAAATCTGGCTCCGAGCTGGGTAAACAAGCAAAAGACATTATGGATGCTGGCAAACTGGTCACTGA
CGAACTGGTGATCGCGCTGGTTAAAGAGCGCATTGCTCAGGAAGACTGCCGTAATGGTTTCCTGTTGGACGGCTT
CCCGCGTACCATTCCGCAGGCAGACGCGATGAAAGAAGCGGGCATCAATGTTGATTACGTTCTGGAATTCGACGT
ACCGGACGAACTGATCGTTGACCGTATCGTCGGTCGCCGCGTTCACGCGCCGTCTGGTCGTGTTTATCACGTTAA
ATTCAATCCGCCGAAAGTAGAAGGCAAAGACGACGTTACCGGTGAAGAACTGACTACCCGTAAAGACGATCAGGA
AGAAACCGTACGTAAACGTCTGGTTGAATACCATCAGATGACTGCACCGCTGATCGGCTACTACTCCAAAGAAGC
GGAAGCGGGTA
>fumC37
CGAGCGCCATTCGTCAGGCGGCGGATGAAGTACTGGCAGGACAGCATGACGACGAATTCCCGCTGGCTATCTGGC
AGACCGGCTCCGGCACGCAAAGTAATATGAACATGAACGAAGTGCTGGCTAACCGGGCCAGTGAATTACTCGGCG
GCGTGCGTGGGATGGAGCGTAAAGTTCACCCTAACGACGACGTGAACAAAAGCCAAAGTTCTAACGATGTCTTTC
CAACGGCGATGCACGTTGCGGCGCTGCTGGCGCTGCGCAAGCAACTCATTCCGCAGCTTAAAACCCTGACACAGA
CACTGAATGAGAAATCGCGCGCATTTGCCGATATCGTCAAAATCGGTCGAACCCACTTGCAGGATGCCACGCCGC
TAACACTGGGGCAGGAGATTTCCGGCTGGGTAGCGATGCTCGAGCATAATCTCAAACATATCGAATACAGCCTGC
CGCATGTAGCGGAACTGGC
>gyrB29
GGTCTGCACGGCGTTGGTGTTTCGGTAGTAAACGCCCTGTCGCAAAAACTGGAGCTGGTTATCCAGCGCGAGGGT
AAAATTCACCGTCAGATCTACGAACACGGTGTACCGCAGGCTCCGCTGGCGGTTACCGGCGAGACTGAAAAAACC
GGCACCATGGTGCGTTTCTGGCCCAGCCTCGAAACCTTCACCAATGTGACCGAGTTCGAATATGAAATTCTGGCG
AAACGTCTGCGTGAGTTGTCGTTCCTCAACTCCGGCGTTTCCATTCGTCTGCGCGACAAGCGCGACGGTAAAGAA
GACCACTTCCACTATGAAGGCGGCATCAAGGCGTTCGTTGAATATCTGAACAAGAACAAAACGCCGATCCACCCG
AATATCTTCTACTTCTCCACCGAAAAAGATGGTATCGGCGTCGAAGTGGCGTTGCAGTGGAACGATGGCTTCCAG
GAAAACATCT
>icd25
CGACGCTGCAGTCGAGAAAGCCTATAAAGGCGAGCGTAAAATCTCCTGGATGGAAATTTACACCGGTGAAAAATC
CACACAGGTTTATGGTCAGGATGTCTGGCTGCCTGCTGAAACCCTTGATCTGATTCGTGAATATCGCGTTGCCAT
TAAAGGCCCTCTGACCACTCCGGTTGGTGGCGGTATTCGTTCTCTGAACGTTGCCCTGCGCCAGGAACTGGATCT
CTACATCTGCCTGCGTCCGGTACGTTACTATCAGGGCACCCCAAGCCCGGTTAAACATCCTGAACTGACCGATAT
GGTTATCTTCCGTGAAAACTCGGAAGACATTTATGCGGGTATCGAATGGAAAGCTGACTCTGCCGACGCCGAGAA
AGTGATTAAATTCCTGCGTGAAGAGATGGGCGTGAAGAAAATTCGCTTCCCGGAACATTGCGGTATCGGTATTAA
GCCGTGTTCGGAAGAAGGCACCAAACGTCTGGTTCGTGCAGCGATCGAATACGCAATTGCTAACGATC
>mdh4
GGTGTAGCGCGTAAACCGGGTATGGATCGTTCCGACCTGTTTAACGTTAACGCCGGCATCGTGAAAAACCTGGTA
CAGCAAGTTGCGAAAACCTGCCCGAAAGCGTGCATTGGTATTATCACTAACCCGGTTAACACCACAGTTGCGATT
GCTGCTGAAGTGCTGAAAAAAGCCGGTGTTTATGACAAAAACAAACTGTTCGGCGTTACCACGCTGGATATCATT
CGTTCCAACACCTTTGTTGCGGAACTGAAAGGCAAACAGCCAGGCGAAGTTGAAGTGCCGGTTATTGGTGGTCAC
TCTGGTGTTACCATTCTGCCGCTGCTGTCACAGGTTCCTGGCGTTAGCTTTACCGAGCAGGAAGTGGCTGATCTG
ACCAAACGTATCCAGAACGCGGGTACTGAGGTGGTTGAAGCGAAAGCCGGTGGCGGGTCTGCAACCCTGTCTATG
GG
>purA5
ATAACGCGCGTGAGAAAGCGCGTGGCGCGAAAGCGATCGGCACCACCGGTCGTGGTATCGGGCCTGCTTATGAAG
ATAAAGTGGCACGTCGCGGTCTGCGTGTTGGCGACCTTTTCGACAAAGAAACCTTCGCTGAAAAACTGAAAGAAG
TGATGGAATATCACAACTTCCAGTTGGTTAACTACTACAAAGCTGAAGCGGTTGATTACCAGAAAGTTCTGGATG
ATACGATGGCTGTTGCCGACATCCTGACTTCTATGGTGGTTGACGTTTCTGACCTGCTCGACCAGGCGCGTCAGC
GTGGCGATTTCGTCATGTTCGAAGGTGCTCAGGGTACGCTGCTGGATATCGACCACGGTACTTATCCGTACGTAA
CATCTTCCAACACCACTGCTGGTGGCGTGGCGACCGGTTCCGGCCTGGGCCCGCGTTATGTTGATTACGTTCTGG
GTATCCTCAAAGCTTACTCCACTCGTGT
>recA73
TGCACGTAAACTGGGCGTCGATATCGACAACCTGCTGTGCTCCCAGCCGGACACTGGCGAGCAGGCACTGGAAAT
CTGTGACGCCCTGGCGCGTTCTGGTGCAGTAGACGTTATCGTCGTTGACTCCGTGGCGGCACTGACGCCGAAAGC
GGAAATCGAAGGCGAAATCGGCGACTCTCACATGGGCCTTGCGGCACGTATGATGAGCCAGGCGATGCGTAAGCT
GGCGGGTAACCTGAAGCAGTCCAACACGCTGCTGATCTTCATCAACCAGATCCGTATGAAAATTGGTGTGATGTT

CGGTAACCCGGAAACCACTACCGGTGGTAACGCGCTGAAATTCTACGCCTCTGTTCGTCTCGACATCCGTCGTAT
CGGCGCGGTGAAAGAGGGCGAAAACGTGGTGGGTAGCGAAACCCGTGTGAAAGTGGTGAAGAACAAAATCGCTGC
TCCGTTTAAACAGGCTGAATTCCAGATCCT
CTACGGCGAAGGTATCAACTTCTATGGCGA

ST58
>adk6
GGGGAAAGGGACTCAGGCTCAGTTCATCATGGAGAAATATGGTATTCCGCAAATCTCCACTGGCGATATGCTGCG
TGCTGCGGTCAAATCTGGCTCCGAGCTGGGTAAACAAGCAAAAGACATTATGGATGCTGGCAAACTGGTCACCGA
CGAACTGGTGATCGCGCTGGTTAAAGAGCGCATTGCTCAGGAAGACTGCCGTAATGGTTTCCTGTTGGACGGCTT
CCCGCGTACCATTCCGCAGGCAGACGCGATGAAAGAAGCGGGCATCAATGTTGATTACGTTCTGGAATTCGACGT
ACCGGACGAACTGATTGTTGATCGTATCGTAGGCCGCCGCGTTCATGCGCCGTCTGGTCGTGTTTATCACGTTAA
ATTCAATCCGCCGAAAGTAGAAGGCAAAGACGACGTTACCGGTGAAGAACTGACTACCCGTAAAGACGATCAGGA
AGAAACCGTGCGTAAACGTCTGGTTGAATACCATCAGATGACTGCACCGCTGATCGGCTACTACTCCAAAGAAGC
GGAAGCGGGTA
>fumC4
CGAGCGCCATTCGGCAGGCGGCGGATGAAGTACTGGCAGGACAGCATGACGACGAATTCCCGCTGGCTATCTGGC
AGACCGGCTCCGGCACGCAAAGTAACATGAACATGAACGAAGTGCTGGCTAACCGGGCCAGTGAATTACTCGGCG
GCGTGCGCGGGATGGAACGTAAAGTTCACCCTAACGACGACGTGAACAAAAGCCAAAGTTCCAACGATGTCTTTC
CGACGGCGATGCACGTTGCGGCGCTGCTGGCGCTGCGCAAGCAACTCATTCCGCAGCTTAAAACCCTGACACAGA
CACTGAGTGAAAAATCGCGTGCATTTGCCGATATCGTCAAAATCGGTCGAACCCACTTGCAGGACGCCACGCCGC
TAACACTAGGGCAGGAGATTTCCGGCTGGGTAGCGATGCTCGAGCATAATCTCAAACATATCGAATACAGCCTGC
CTCACGTAGCGGAACTGGC
>gyrB4
GGTCTGCACGGCGTTGGTGTTTCGGTAGTAAACGCCCTGTCGCAAAAACTGGAGCTGGTTATCCAGCGCGAGGGT
AAAATTCACCGTCAGATCTACGAACACGGTGTACCGCAGGCCCCGCTGGCGGTTACCGGCGAGACTGAAAAAACC
GGCACCATGGTGCGTTTCTGGCCCAGCCTCGAAACCTTCACCAATGTGACCGAGTTCGAATATGAAATTCTGGCG
AAACGTCTGCGTGAGTTGTCGTTCCTCAACTCCGGCGTTTCCATTCGTCTGCGCGACAAGCGCGACGGCAAAGAA
GACCACTTCCACTATGAAGGCGGCATCAAGGCGTTCGTTGAATATCTGAACAAGAACAAAACGCCGATCCACCCG
AATATCTTCTACTTCTCCACTGAAAAAGACGGTATTGGCGTCGAAGTGGCGTTGCAGTGGAACGATGGCTTCCAG
GAAAACATCT
>icd16
CGACGCTGCAGTCGAGAAAGCCTATAAAGGCGAGCGTAAAATCTCCTGGATGGAAATTTACACCGGTGAAAAATC
CACACAGGTTTATGGTCAGGACGTCTGGCTGCCTGCTGAAACTCTTGATCTGATTCGTGAATATCGCGTTGCCAT
TAAAGGTCCGCTGACCACTCCTGTTGGTGGCGGTATTCGCTCTCTGAACGTTGCCCTGCGCCAGGAACTGGATCT
CTACATCTGCCTGCGTCCGGTACGTTACTATCAAGGCACTCCAAGCCCGGTTAAACACCCTGAACTGACCGATAT
GGTTATCTTCCGTGAAAACTCGGAAGACATTTATGCGGGTATCGAATGGAAAGCAGACTCTGCCGACGCCGAGAA
AGTGATTAAATTCCTGCGTGAAGAGATGGGGGTGAAGAAAATTCGCTTCCCGGAACATTGTGGTATCGGTATTAA
GCCGTGTTCGGAAGAAGGCACCAAACGTCTGGTTCGTGCAGCGATCGAATACGCAATTGCTAACGATC
>mdh24
GGTGTAGCGCGTAAACCGGGTATGGATCGTTCCGACCTGTTTAACGTTAACGCCGGCATCGTGAAAAACCTGGTA
CAGCAAGTTGCGAAAACCTGCCCGAAAGCGTGCATTGGTATTATCACTAACCCGGTTAACACCACTGTTGCAATT
GCTGCTGAAGTGCTGAAAAAAGCCGGTGTTTATGACAAAAACAAACTGTTCGGCGTTACCACGCTGGATATCATT
CGTTCCAACACCTTTGTTGCGGAACTGAAAGGCAAACAGCCAGGCGAAGTTGAAGTGCCGGTTATTGGCGGTCAC
TCTGGTGTTACCATTCTGCCGCTGCTGTCACAGGTTCCTGGCGTTAGTTTTACCGAGCAGGAAGTGGCTGATCTG
ACCAAACGTATCCAGAACGCGGGTACTGAGGTGGTTGAAGCGAAAGCCGGTGGCGGGTCTGCAACCCTGTCTATG
GG
>purA8
ATAACGCGCGTGAGAAAGCGCGTGGCGCGAAAGCGATCGGCACCACCGGTCGTGGTATCGGGCCTGCTTATGAAG
ATAAAGTAGCACGTCGCGGTCTGCGTGTTGGCGACCTTTTCGACAAAGAAACCTTCGCTGAAAAACTGAAAGAAG
TGATGGAATATCACAACTTCCAGTTGGTTAACTACTACAAAGCTGAAGCGGTTGATTACCAGAAAGTTCTGGATG
ATACGATGGCTGTTGCCGACATCCTGACTTCTATGGTGGTTGACGTTTCTGACCTGCTCGACCAGGCGCGTCAGC
GTGGCGATTTCGTCATGTTTGAAGGTGCGCAGGGTACGCTGCTGGATATCGACCACGGTACTTATCCGTACGTAA
CTTCTTCCAACACCACTGCTGGTGGCGTGGCGACCGGTTCCGGCCTGGGCCCGCGTTATGTTGATTACGTTCTGG
GTATCCTCAAAGCTTACTCCACTCGTGT
>recA14
CGCACGTAAACTGGGCGTCGATATTGATAACCTGCTGTGCTCCCAGCCGGACACCGGCGAGCAGGCACTGGAAAT
CTGTGACGCCCTGGCGCGTTCTGGCGCAGTAGACGTTATCGTCGTTGACTCCGTGGCGGCACTGACGCCGAAAGC
GGAAATCGAAGGCGAAATCGGCGACTCTCACATGGGCCTTGCGGCACGTATGATGAGCCAGGCGATGCGTAAGCT
GGCGGGTAACCTGAAGCAGTCCAACACGCTGCTGATCTTCATCAACCAGATCCGTATGAAAATTGGTGTGATGTT

CGGTAACCCGGAAACCACTACCGGTGGTAACGCGCTGAAATTCTACGCCTCTGTTCGTCTCGACATCCGTCGTAT
CGGCGCGGTGAAAGAGGGCGAAAACGTGGTGGGTAGCGAAACCCGCGTGAAAGTGGTGAAGAACAAAATCGCTGC
ACCGTTTAAACAGGCTGAATTTCAGATCCTCTACGGCGAAGGTATCAACTTCTACGGCGA

ST10

>adk10
GGGGAAAGGGACTCAGGCTCAGTTCATCATGGAGAAATATGGTATTCCGCAAATCTCCACTGGCGATATGCTGCG
TGCTGCGGTCAAATCTGGCTCCGAGCTGGGTAAACAAGCAAAAGACATTATGGATGCTGGCAAACTGGTCACCGA
CGAACTGGTGATCGCGCTGGTTAAAGAGCGCATTGCTCAGGAAGACTGCCGTAATGGTTTCCTGTTGGACGGCTT
CCCGCGTACCATTCCGCAGGCAGACGCGATGAAAGAAGCGGGCATCAATGTTGATTACGTTCTGGAATTCGACGT
ACCGGACGAACTGATCGTTGACCGTATCGTCGGTCGCCGCGTTCATGCGCCGTCTGGTCGTGTTTATCACGTTAA
ATTCAATCCGCCGAAAGTAGAAGGCAAAGACGACGTTACCGGTGAAGAACTGACTACCCGTAAAGATGATCAGGA
AGAGACCGTACGTAAACGTCTGGTTGAATACCATCAGATGACAGCACCGCTGATCGGCTACTACTCCAAAGAAGC
AGAAGCGGGTA
>fumC11
CGAGCGCCATTCGTCAGGCGGCGGATGAAGTACTGGCAGGACAGCATGACGACGAATTCCCGCTGGCTATCTGGC
AGACCGGCTCCGGCACGCAAAGTAACATGAACATGAACGAAGTGCTGGCTAACCGGGCCAGTGAATTACTCGGCG
GTGTGCGCGGGATGGAACGTAAAGTTCACCCTAACGACGACGTGAACAAAAGCCAAAGTTCCAACGATGTCTTTC
CGACGGCGATGCACGTTGCGGCGCTGCTGGCGCTGCGCAAGCAACTCATTCCTCAGCTTAAAACCCTGACACAGA
CACTGAATGAGAAATCCCGTGCTTTTGCCGATATCGTCAAAATTGGTCGTACTCACTTGCAGGATGCCACGCCGT
TAACGCTGGGGCAGGAGATTTCCGGCTGGGTAGCGATGCTCGAGCATAATCTCAAACATATCGAATACAGCCTGC
CTCACGTAGCGGAACTGGC
>gyrB4
GGTCTGCACGGCGTTGGTGTTTCGGTAGTAAACGCCCTGTCGCAAAAACTGGAGCTGGTTATCCAGCGCGAGGGT
AAAATTCACCGTCAGATCTACGAACACGGTGTACCGCAGGCCCCGCTGGCGGTTACCGGCGAGACTGAAAAAACC
GGCACCATGGTGCGTTTCTGGCCCAGCCTCGAAACCTTCACCAATGTGACCGAGTTCGAATATGAAATTCTGGCG
AAACGTCTGCGTGAGTTGTCGTTCCTCAACTCCGGCGTTTCCATTCGTCTGCGCGACAAGCGCGACGGCAAAGAA
GACCACTTCCACTATGAAGGCGGCATCAAGGCGTTCGTTGAATATCTGAACAAGAACAAAACGCCGATCCACCCG
AATATCTTCTACTTCTCCACTGAAAAAGACGGTATTGGCGTCGAAGTGGCGTTGCAGTGGAACGATGGCTTCCAG
GAAAACATCT
>icd8
CGACGCTGCAGTCGAGAAAGCCTATAAAGGCGAGCGTAAAATCTCCTGGATGGAAATTTACACCGGTGAAAAATC
CACACAGGTTTATGGTCAGGACGTCTGGCTGCCTGCTGAAACTCTTGATCTGATTCGTGAATATCGCGTTGCCAT
TAAAGGTCCGCTGACCACTCCGGTTGGTGGCGGTATTCGCTCTCTGAACGTTGCCCTGCGCCAGGAACTGGATCT
CTACATCTGCCTGCGTCCGGTACGTTACTATCAGGGCACTCCAAGCCCGGTTAAACACCCTGAACTGACCGATAT
GGTTATCTTCCGTGAAAACTCGGAAGACATTTATGCGGGTATCGAATGGAAAGCAGACTCTGCCGACGCCGAGAA
AGTGATTAAATTCCTGCGTGAAGAGATGGGGGTGAAGAAAATTCGCTTCCCGGAACATTGTGGTATCGGTATTAA
GCCGTGTTCGGAAGAAGGCACCAAACGTCTGGTTCGTGCAGCGATCGAATACGCAATTGCTAACGATC
>mdh8
GGCGTAGCGCGTAAACCGGGTATGGATCGTTCCGACCTGTTTAACGTTAACGCCGGCATCGTGAAAAACCTGGTA
CAGCAAGTTGCGAAAACCTGCCCGAAAGCGTGCATTGGTATTATCACTAACCCGGTTAACACCACAGTTGCAATT
GCTGCTGAAGTGCTGAAAAAAGCCGGTGTTTATGACAAAAACAAACTGTTCGGCGTTACCACGCTGGATATCATT
CGTTCCAACACCTTTGTTGCGGAACTGAAAGGCAAACAGCCAGGCGAAGTTGAAGTGCCGGTTATTGGCGGTCAC
TCTGGTGTTACCATTCTGCCGCTGCTGTCACAGGTTCCTGGCGTTAGTTTTACCGAGCAGGAAGTGGCTGATCTG
ACCAAACGCATCCAGAACGCGGGTACTGAAGTGGTTGAAGCGAAGGCCGGTGGCGGGTCTGCAACCCTGTCTATG
GG
>purA8
ATAACGCGCGTGAGAAAGCGCGTGGCGCGAAAGCGATCGGCACCACCGGTCGTGGTATCGGGCCTGCTTATGAAG
ATAAAGTAGCACGTCGCGGTCTGCGTGTTGGCGACCTTTTCGACAAAGAAACCTTCGCTGAAAAACTGAAAGAAG
TGATGGAATATCACAACTTCCAGTTGGTTAACTACTACAAAGCTGAAGCGGTTGATTACCAGAAAGTTCTGGATG
ATACGATGGCTGTTGCCGACATCCTGACTTCTATGGTGGTTGACGTTTCTGACCTGCTCGACCAGGCGCGTCAGC
GTGGCGATTTCGTCATGTTTGAAGGTGCGCAGGGTACGCTGCTGGATATCGACCACGGTACTTATCCGTACGTAA
CTTCTTCCAACACCACTGCTGGTGGCGTGGCGACCGGTTCCGGCCTGGGCCCGCGTTATGTTGATTACGTTCTGG
GTATCCTCAAAGCTTACTCCACTCGTGT
>recA2
CGCACGTAAACTGGGCGTCGATATCGACAACCTGCTGTGCTCCCAGCCGGACACCGGCGAGCAGGCACTGGAAAT
CTGTGACGCCCTGGCGCGTTCTGGCGCAGTAGACGTTATCGTCGTTGACTCCGTGGCGGCACTGACGCCGAAAGC
GGAAATCGAAGGCGAAATCGGCGACTCTCACATGGGCCTTGCGGCACGTATGATGAGCCAGGCGATGCGTAAGCT
GGCGGGTAACCTGAAGCAGTCCAACACGCTGCTGATCTTCATCAACCAGATCCGTATGAAAATTGGTGTGATGTT

CGGTAACCCGGAAACCACTACCGGTGGTAACGCGCTGAAATTCTACGCCTCTGTTCGTCTCGACATCCGTCGTAT
CGGCGCGGTGAAAGAGGGCGAAAACGTGGTGGGTAGCGAAACCCGCGTGAAAGTGGTGAAGAACAAAATCGCTGC
GCCGTTTAAACAGGCTGAATTCCAGATCCTCTACGGCGAAGGTATCAACTTCTACGGCGA

ST155
>adk6
GGGGAAAGGGACTCAGGCTCAGTTCATCATGGAGAAATATGGTATTCCGCAAATCTCCACTGGCGATATGCTGCG
TGCTGCGGTCAAATCTGGCTCCGAGCTGGGTAAACAAGCAAAAGACATTATGGATGCTGGCAAACTGGTCACCGA
CGAACTGGTGATCGCGCTGGTTAAAGAGCGCATTGCTCAGGAAGACTGCCGTAATGGTTTCCTGTTGGACGGCTT
CCCGCGTACCATTCCGCAGGCAGACGCGATGAAAGAAGCGGGCATCAATGTTGATTACGTTCTGGAATTCGACGT
ACCGGACGAACTGATTGTTGATCGTATCGTAGGCCGCCGCGTTCATGCGCCGTCTGGTCGTGTTTATCACGTTAA
ATTCAATCCGCCGAAAGTAGAAGGCAAAGACGACGTTACCGGTGAAGAACTGACTACCCGTAAAGACGATCAGGA
AGAAACCGTGCGTAAACGTCTGGTTGAATACCATCAGATGACTGCACCGCTGATCGGCTACTACTCCAAAGAAGC
GGAAGCGGGTA
>fumC4
CGAGCGCCATTCGGCAGGCGGCGGATGAAGTACTGGCAGGACAGCATGACGACGAATTCCCGCTGGCTATCTGGC
AGACCGGCTCCGGCACGCAAAGTAACATGAACATGAACGAAGTGCTGGCTAACCGGGCCAGTGAATTACTCGGCG
GCGTGCGCGGGATGGAACGTAAAGTTCACCCTAACGACGACGTGAACAAAAGCCAAAGTTCCAACGATGTCTTTC
CGACGGCGATGCACGTTGCGGCGCTGCTGGCGCTGCGCAAGCAACTCATTCCGCAGCTTAAAACCCTGACACAGA
CACTGAGTGAAAAATCGCGTGCATTTGCCGATATCGTCAAAATCGGTCGAACCCACTTGCAGGACGCCACGCCGC
TAACACTAGGGCAGGAGATTTCCGGCTGGGTAGCGATGCTCGAGCATAATCTCAAACATATCGAATACAGCCTGC
CTCACGTAGCGGAACTGGC
>gyrB14
GGTCTGCACGGCGTTGGTGTTTCGGTAGTAAACGCCCTGTCGCAAAAACTGGAGCTGGTTATCCAGCGCGAGGGT
AAAATTCACCGTCAGATCTACGAACACGGTGTACCGCAGGCCCCGCTGGCGGTTACCGGCGAGACTGAAAAAACC
GGCACCATGGTGCGTTTCTGGCCTAGCCTCGAAACCTTCACCAATGTGACCGAGTTCGAATATGAAATTCTGGCG
AAACGTCTGCGTGAGTTGTCGTTCCTCAACTCCGGCGTTTCCATTCGTCTGCGCGACAAGCGTGACGGCAAAGAA
GACCACTTCCACTATGAAGGCGGCATCAAGGCGTTCGTTGAATATCTGAACAAGAACAAAACGCCGATCCACCCG
AATATCTTCTACTTCTCCACCGAAAAAGACGGTATTGGCGTCGAAGTGGCGTTGCAGTGGAACGATGGCTTCCAG
GAAAACATCT
>icd16
CGACGCTGCAGTCGAGAAAGCCTATAAAGGCGAGCGTAAAATCTCCTGGATGGAAATTTACACCGGTGAAAAATC
CACACAGGTTTATGGTCAGGACGTCTGGCTGCCTGCTGAAACTCTTGATCTGATTCGTGAATATCGCGTTGCCAT
TAAAGGTCCGCTGACCACTCCTGTTGGTGGCGGTATTCGCTCTCTGAACGTTGCCCTGCGCCAGGAACTGGATCT
CTACATCTGCCTGCGTCCGGTACGTTACTATCAAGGCACTCCAAGCCCGGTTAAACACCCTGAACTGACCGATAT
GGTTATCTTCCGTGAAAACTCGGAAGACATTTATGCGGGTATCGAATGGAAAGCAGACTCTGCCGACGCCGAGAA
AGTGATTAAATTCCTGCGTGAAGAGATGGGGGTGAAGAAAATTCGCTTCCCGGAACATTGTGGTATCGGTATTAA
GCCGTGTTCGGAAGAAGGCACCAAACGTCTGGTTCGTGCAGCGATCGAATACGCAATTGCTAACGATC
>mdh24
GGTGTAGCGCGTAAACCGGGTATGGATCGTTCCGACCTGTTTAACGTTAACGCCGGCATCGTGAAAAACCTGGTA
CAGCAAGTTGCGAAAACCTGCCCGAAAGCGTGCATTGGTATTATCACTAACCCGGTTAACACCACTGTTGCAATT
GCTGCTGAAGTGCTGAAAAAAGCCGGTGTTTATGACAAAAACAAACTGTTCGGCGTTACCACGCTGGATATCATT
CGTTCCAACACCTTTGTTGCGGAACTGAAAGGCAAACAGCCAGGCGAAGTTGAAGTGCCGGTTATTGGCGGTCAC
TCTGGTGTTACCATTCTGCCGCTGCTGTCACAGGTTCCTGGCGTTAGTTTACCGAGCAGGAAGTGGCTGATCTG
ACCAAACGTATCCAGAACGCGGGTACTGAGGTGGTTGAAGCGAAAGCCGGTGGCGGGTCTGCAACCCTGTCTATG
GG

>purA8
ATAACGCGCGTGAGAAAGCGCGTGGCGCGAAAGCGATCGGCACCACCGGTCGTGGTATCGGGCCTGCTTATGAAG
ATAAAGTAGCACGTCGCGGTCTGCGTGTTGGCGACCTTTTCGACAAAGAAACCTTCGCTGAAAAACTGAAAGAAG
TGATGGAATATCACAACTTCCAGTTGGTTAACTACTACAAAGCTGAAGCGGTTGATTACCAGAAAGTTCTGGATG
ATACGATGGCTGTTGCCGACATCCTGACTTCTATGGTGGTTGACGTTTCTGACCTGCTCGACCAGGCGCGTCAGC
GTGGCGATTTCGTCATGTTTGAAGGTGCGCAGGGTACGCTGCTGGATATCGACCACGGTACTTATCCGTACGTAA
CTTCTTCCAACACCACTGCTGGTGGCGTGGCGACCGGTTCCGGCCTGGGCCCGCGTTATGTTGATTACGTTCTGG
GTATCCTCAAAGCTTACTCCACTCGTGT
>recA14
CGCACGTAAACTGGGCGTCGATATTGATAACCTGCTGTGCTCCCAGCCGGACACCGGCGAGCAGGCACTGGAAAT
CTGTGACGCCCTGGCGCGTTCTGGCGCAGTAGACGTTATCGTCGTTGACTCCGTGGCGGCACTGACGCCGAAAGC
GGAAATCGAAGGCGAAATCGGCGACTCTCACATGGGCCTTGCGGCACGTATGATGAGCCAGGCGATGCGTAAGCT
GGCGGGTAACCTGAAGCAGTCCAACACGCTGCTGATCTTCATCAACCAGATCCGTATGAAAATTGGTGTGATGTT

CGGTAACCCGGAAACCACTACCGGTGGTAACGCGCTGAAATTCTACGCCTCTGTTCGTCTCGACATCCGTCGTAT
CGGCGCGGTGAAAGAGGGCGAAAACGTGGTGGGTAGCGAAACCCGCGTGAAAGTGGTGAAGAACAAAATCGCTGC
ACCGTTTAAACAGGCTGAATTTCAGATCCTCTACGGCGAAGGTATCAACTTCTACGGCGA

ST399

>adk6
GGGGAAAGGGACTCAGGCTCAGTTCATCATGGAGAAATATGGTATTCCGCAAATCTCCACTGGCGATATGCTGCG
TGCTGCGGTCAAATCTGGCTCCGAGCTGGGTAAACAAGCAAAAGACATTATGGATGCTGGCAAACTGGTCACCGA
CGAACTGGTGATCGCGCTGGTTAAAGAGCGCATTGCTCAGGAAGACTGCCGTAATGGTTTCCTGTTGGACGGCTT
CCCGCGTACCATTCCGCAGGCAGACGCGATGAAAGAAGCGGGCATCAATGTTGATTACGTTCTGGAATTCGACGT
ACCGGACGAACTGATTGTTGATCGTATCGTAGGCCGCCGCGTTCATGCGCCGTCTGGTCGTGTTTATCACGTTAA
ATTCAATCCGCCGAAAGTAGAAGGCAAAGACGACGTTACCGGTGAAGAACTGACTACCCGTAAAGACGATCAGGA
AGAAACCGTGCGTAAACGTCTGGTTGAATACCATCAGATGACTGCACCGCTGATCGGCTACTACTCCAAAGAAGC
GGAAGCGGGTA
>fumC4
CGAGCGCCATTCGGCAGGCGGCGGATGAAGTACTGGCAGGACAGCATGACGACGAATTCCCGCTGGCTATCTGGC
AGACCGGCTCCGGCACGCAAAGTAACATGAACATGAACGAAGTGCTGGCTAACCGGGCCAGTGAATTACTCGGCG
GCGTGCGCGGGATGGAACGTAAAGTTCACCCTAACGACGACGTGAACAAAAGCCAAAGTTCCAACGATGTCTTTC
CGACGGCGATGCACGTTGCGGCGCTGCTGGCGCTGCGCAAGCAACTCATTCCGCAGCTTAAAACCCTGACACAGA
CACTGAGTGAAAAATCGCGTGCATTTGCCGATATCGTCAAAATCGGTCGAACCCACTTGCAGGACGCCACGCCGC
TAACACTAGGGCAGGAGATTTCCGGCTGGGTAGCGATGCTCGAGCATAATCTCAAACATATCGAATACAGCCTGC
CTCACGTAGCGGAACTGGC
>gyrB1
GGTCTGCACGGCGTTGGTGTTTCGGTAGTAAACGCCCTGTCGCAAAAACTGGAGCTGGTTATCCAGCGCGAGGGT
AAAATTCACCGTCAGATCTACGAACACGGTGTACCGCAGGCTCCGCTGGCGGTTACCGGCGAGACTGAAAAAACC
GGCACCATGGTGCGTTTCTGGCCCAGCCTCGAAACCTTCACCAATGTGACCGAGTTCGAATATGAAATTCTGGCG
AAACGTCTGCGTGAGTTGTCGTTCCTCAACTCCGGCGTTTCCATTCGTCTGCGCGACAAGCGTGACGGCAAAGAA
GACCACTTCCACTATGAAGGCGGCATCAAGGCGTTCGTTGAATATCTGAACAAGAACAAAACGCCGATCCATCCG
AATATCTTCTACTTCTCCACCGAAAAAGACGGTATTGGCGTCGAAGTGGCGTTGCAGTGGAACGATGGCTTCCAG
GAAAACATCT
>icd95
CGACGCTGCAGTCGAGAAAGCCTATAAAGGCGAGCGTAAAATCTCCTGGATGGAAATTTACACCGGTGAAAAATC
CACACAGGTTTATGGTCAGGATGTCTGGCTGCCTGCTGAAACCCTTGATCTGATTCGTGAATATCGCGTTGCCAT
TAAAGGTCCGCTGACCACTCCTGTTGGTGGCGGTATTCGCTCTCTGAACGTTGCCCTGCGCCAGGAACTGGATCT
CTACATCTGCCTGCGTCCGGTACGTTACTATCAGGGCACTCCAAGCCCGGTTAAACACCCTGAACTGACCGATAT
GGTTATTTTCCGTGAAAACTCGGAAGACATTTATGCGGGTATCGAATGGAAAGCAGACTCTGCCGACGCCGAGAA
AGTAATTAAATTCCTGCGTGAAGAGATGGGGGTGAAGAAAATTCGCTTCCCGGAACATTGTGGTATCGGTATTAA
GCCGTGTTCGGAAGAAGGCACCAAACGTCTGGTTCGTGCAGCGATCGAATACGCAATTGCTAACGATC
>mdh69
GGTGTAGCGCGTAAACCGGGTATGGATCGTTCCGACCTGTTTAACGTTAACGCCGGCATCGTGAAAAACCTGGTA
CAGCAAGTTGCGAAAACCTGCCCGAAAGCGTGTATTGGTATTATCACTAACCCGGTTAACACCACAGTTGCGATT
GCTGCTGAAGTGCTGAAAAAAGCCGGTGTTTATGACAAAAACAAACTGTTCGGCGTTACCACGCTGGATATCATT
CGTTCCAACACTTTTGTTGCGGAACTGAAAGGCAAACAGCCAGGCGAAGTTGAAGTGCCGGTTATTGGCGGTCAC
TCTGGTGTTACCATTCTGCCGCTGCTGTCACAGGTTCCTGGCGTTAGTTTTACCGAGCAGGAAGTGGCTGATCTG
ACCAAACGTATCCAGAACGCGGGTACTGAGGTGGTTGAAGCGAAAGCCGGTGGCGGGTCTGCAACCCTGTCTATG
GG
>purA8
ATAACGCGCGTGAGAAAGCGCGTGGCGCGAAAGCGATCGGCACCACCGGTCGTGGTATCGGGCCTGCTTATGAAG
ATAAAGTAGCACGTCGCGGTCTGCGTGTTGGCGACCTTTTCGACAAAGAAACCTTCGCTGAAAAACTGAAAGAAG
TGATGGAATATCACAACTTCCAGTTGGTTAACTACTACAAAGCTGAAGCGGTTGATTACCAGAAAGTTCTGGATG
ATACGATGGCTGTTGCCGACATCCTGACTTCTATGGTGGTTGACGTTTCTGACCTGCTCGACCAGGCGCGTCAGC
GTGGCGATTTCGTCATGTTTGAAGGTGCGCAGGGTACGCTGCTGGATATCGACCACGGTACTTATCCGTACGTAA
CTTCTTCCAACACCACTGCTGGTGGCGTGGCGACCGGTTCCGGCCTGGGCCCGCGTTATGTTGATTACGTTCTGG
GTATCCTCAAAGCTTACTCCACTCGTGT
>recA20
CGCACGTAAACTGGGCGTCGATATCGATAACCTGCTGTGCTCCCAGCCGGACACCGGCGAGCAGGCACTGGAAAT
CTGTGACGCCCTGGCGCGTTCTGGCGCAGTAGACGTTATCGTCGTTGACTCCGTGGCGGCACTGACGCCGAAAGC
GGAAATCGAAGGCGAAATCGGCGACTCTCACATGGGCCTTGCGGCACGTATGATGAGCCAGGCGATGCGTAAGCT
GGCGGGTAACCTGAAGCAGTCCAACACGCTGCTGATCTTCATCAACCAGATCCGTATGAAAATTGGTGTAATGTT

CGGTAACCCGGAAACCACTACCGGTGGTAACGCGCTGAAATTCTACGCCTCTGTTCGTCTCGACATCCGTCGTAT
CGGCGCGGTGAAAGAGGGCGAAAACGTGGTGGGTAGCGAAACCCGCGTGAAAGTGGTGAAGAACAAAATCGCTGC
GCCGTTTAAACAGGCTGAATTCCAGATCCTCTACGGCGAAGGTATCAACTTCTACGGCGA

ST617

>adk10
GGGGAAAGGGACTCAGGCTCAGTTCATCATGGAGAAATATGGTATTCCGCAAATCTCCACTGGCGATATGCTGCG
TGCTGCGGTCAAATCTGGCTCCGAGCTGGGTAAACAAGCAAAAGACATTATGGATGCTGGCAAACTGGTCACCGA
CGAACTGGTGATCGCGCTGGTTAAAGAGCGCATTGCTCAGGAAGACTGCCGTAATGGTTTCCTGTTGGACGGCTT
CCCGCGTACCATTCCGCAGGCAGACGCGATGAAAGAAGCGGGCATCAATGTTGATTACGTTCTGGAATTCGACGT
ACCGGACGAACTGATCGTTGACCGTATCGTCGGTCGCCGCGTTCATGCGCCGTCTGGTCGTGTTTATCACGTTAA
ATTCAATCCGCCGAAAGTAGAAGGCAAAGACGACGTTACCGGTGAAGAACTGACTACCCGTAAAGATGATCAGGA
AGAGACCGTACGTAAACGTCTGGTTGAATACCATCAGATGACAGCACCGCTGATCGGCTACTACTCCAAAGAAGC
AGAAGCGGGTA

>fumC11
CGAGCGCCATTCGTCAGGCGGCGGATGAAGTACTGGCAGGACAGCATGACGACGAATTCCCGCTGGCTATCTGGC
AGACCGGCTCCGGCACGCAAAGTAACATGAACATGAACGAAGTGCTGGCTAACCGGGCCAGTGAATTACTCGGCG
GTGTGCGCGGGATGGAACGTAAAGTTCACCCTAACGACGACGTGAACAAAAGCCAAAGTTCCAACGATGTCTTTC
CGACGGCGATGCACGTTGCGGCGCTGCTGGCGCTGCGCAAGCAACTCATTCCTCAGCTTAAAACCCTGACACAGA
CACTGAATGAGAAATCCCGTGCTTTTGCCGATATCGTCAAAATTGGTCGTACTCACTTGCAGGATGCCACGCCGT
TAACGCTGGGGCAGGAGATTTCCGGCTGGGTAGCGATGCTCGAGCATAATCTCAAACATATCGAATACAGCCTGC
CTCACGTAGCGGAACTGGC

>gyrB4
GGTCTGCACGGCGTTGGTGTTTCGGTAGTAAACGCCCTGTCGCAAAAACTGGAGCTGGTTATCCAGCGCGAGGGT
AAAATTCACCGTCAGATCTACGAACACGGTGTACCGCAGGCCCCGCTGGCGGTTACCGGCGAGACTGAAAAAACC
GGCACCATGGTGCGTTTCTGGCCCAGCCTCGAAACCTTCACCAATGTGACCGAGTTCGAATATGAAATTCTGGCG
AAACGTCTGCGTGAGTTGTCGTTCCTCAACTCCGGCGTTTCCATTCGTCTGCGCGACAAGCGCGACGGCAAAGAA
GACCACTTCCACTATGAAGGCGGCATCAAGGCGTTCGTTGAATATCTGAACAAGAACAAAACGCCGATCCACCCG
AATATCTTCTACTTCTCCACTGAAAAAGACGGTATTGGCGTCGAAGTGGCGTTGCAGTGGAACGATGGCTTCCAG
GAAAACATCT

>icd8
CGACGCTGCAGTCGAGAAAGCCTATAAAGGCGAGCGTAAAATCTCCTGGATGGAAATTTACACCGGTGAAAAATC
CACACAGGTTTATGGTCAGGACGTCTGGCTGCCTGCTGAAACTCTTGATCTGATTCGTGAATATCGCGTTGCCAT
TAAAGGTCCGCTGACCACTCCGGTTGGTGGCGGTATTCGCTCTCTGAACGTTGCCCTGCGCCAGGAACTGGATCT
CTACATCTGCCTGCGTCCGGTACGTTACTATCAGGGCACTCCAAGCCCGGTTAAACACCCTGAACTGACCGATAT
GGTTATCTTCCGTGAAAACTCGGAAGACATTTATGCGGGTATCGAATGGAAAGCAGACTCTGCCGACGCCGAGAA
AGTGATTAAATTCCTGCGTGAAGAGATGGGGGTGAAGAAAATTCGCTTCCCGGAACATTGTGGTATCGGTATTAA
GCCGTGTTCGGAAGAAGGCACCAAACGTCTGGTTCGTGCAGCGATCGAATACGCAATTGCTAACGATC

>mdh8
GGCGTAGCGCGTAAACCGGGTATGGATCGTTCCGACCTGTTTAACGTTAACGCCGGCATCGTGAAAAACCTGGTA
CAGCAAGTTGCGAAAACCTGCCCGAAAGCGTGCATTGGTATTATCACTAACCCGGTTAACACCACAGTTGCAATT
GCTGCTGAAGTGCTGAAAAAAGCCGGTGTTTATGACAAAAACAAACTGTTCGGCGTTACCACGCTGGATATCATT
CGTTCCAACACCTTTGTTGCGGAACTGAAAGGCAAACAGCCAGGCGAAGTTGAAGTGCCGGTTATTGGCGGTCAC
TCTGGTGTTACCATTCTGCCGCTGCTGTCACAGGTTCCTGGCGTTAGTTTTACCGAGCAG
GAAGTGGCTGATCTGACCAAACGCATCCAGAACGCGGGTACTGAAGTGGTTGAAGCGAAGGCCGGTGGCGGGTCT
GCAACCCTGTCTATGGG

>purA13
ATAACGCGCGTGAGAAAGCGCGTGGCGCGAAAGCGATCGGCACAACCGGTCGTGGTATCGGGCCTGCTTATGAAG
ATAAAGTAGCACGTCGCGGTCTGCGTGTTGGCGACCTTTTCGACAAAGAAACCTTCGCTGAAAAACTGAAAGAAG
TGATGGAATATCACAACTTCCAGTTGGTTAACTACTACAAAGCTGAAGCGGTTGATTACCAGAAAGTTCTGGATG
ATACGATGGCAGTTGCCGACATCCTGACTTCTATGGTGGTTGACGTTTCTGACCTGCTCGACCAGGCGCGTCAGC
GTGGCGATTTCGTCATGTTTGAAGGTGCGCAGGGTACGCTGCTGGATATCGACCACGGTACTTATCCGTACGTAA
CTTCTTCCAACACCACTGCTGGTGGCGTGGCGACCGGTTCCGGCCTGGGCCCGCGTTATGTTGATTATGTTCTGG
GTATCCTCAAAGCTTACTCAACTCGTGT

>recA73
TGCACGTAAACTGGGCGTCGATATCGACAACCTGCTGTGCTCCCAGCCGGACACTGGCGAGCAGGCACTGGAAAT
CTGTGACGCCCTGGCGCGTTCTGGTGCAGTAGACGTTATCGTCGTTGACTCCGTGGCGGCACTGACGCCGAAAGC
GGAAATCGAAGGCGAAATCGGCGACTCTCACATGGGCCTTGCGGCACGTATGATGAGCCAGGCGATGCGTAAGCT
GGCGGGTAACCTGAAGCAGTCCAACACGCTGCTGATCTTCATCAACCAGATCCGTATGAAAATTGGTGTGATGTT

CGGTAACCCGGAAACCACTACCGGTGGTAACGCGCTGAAATTCTACGCCTCTGTTCGTCTCGACATCCGTCGTAT
CGGCGCGGTGAAAGAGGGCGAAAACGTGGTGGGTAGCGAAACCCGTGTGAAAGTGGTGAAGAACAAAATCGCTGC
TCCGTTTAAACAGGCTGAATTCCAGATCCTCTACGGCGAAGGTATCAACTTCTATGGCGA

Sequences of 7 seven housekeeping genes of *K. pneumoniae*

ST471

>gapA3
AACCTGAAGTGGGACGAAGTTGGTGTTGACGTTGTTGCTGAAGCAACCGGTATCTTCCTGACCGACGAAACCGCT
CGTAAACACATCACCGCTGGCGCGAAAAAAGTCGTTCTGACTGGCCCGTCCAAAGACAACACTCCGATGTTCGTT
CGCGGCGCTAACTTCGACGCTTACGCTGGCCAGGACATCGTTTCCAACGCTTCCTGCACCACTAACTGCCTGGCG
CCGCTGGCTAAAGTTATCAACGACAACTTCGGTATCGTTGAAGGCCTGATGACCACCGTCCACGCTACCACCGCT
ACTCAGAAAACCGTTGATGGCCCGTCTCACAAAGACTGGCGCGGCGGCCGCGGCGCAGCTCAGAACATCATCCCG
TCCTCTACCGGCGCTGCTAAAGCAGTAGGTAAAGTACTGCCAGAACTGAACGGCAAACTGACCGGTATGGCGTTC

>infB1
GGCATGATCACCTTCCTGGATACCCCGGGCCACGCCGCGTTTACCTCCATGCGTGCTCGTGGCGCGCAGGCGACG
GATATCGTGGTTCTGGTGGTGGCGGCAGACGACGGCGTGATGCCGCAGACTATCGAAGCTATCCAGCACGCTAAA
GCGGCGCAGGTACCGGTGGTAGTGGCGGTGAACAAGATCGATAAGCCAGAAGCCGATCCGGATCGCGTGAAGAAC
GAACTGTCCCAGTACGGCATCCTGCCGGAAGAGTGGGGCGGCGAGAGCCAGTTCGTCCACGTTTCCGCGAAAGCG
GGTACCGGCATCGACGAC
>mdh1
GGCGCGGATGTAGTGCTGATCTCCGCGGGCGTGGCGCGTAAGCCCGGCATGGATCGTTCCGACCTGTTTAATGTG
AATGCGGGTATCGTGAAGAACCTCGTGCAGCAGATTGCCAAAACCTGCCCGCAGGCCTGCATCGGCATTATCACC
AACCCGGTGAATACCACCGTGGCTATCGCCGCCGAAGTACTGAAAAAAGCCGGCGTGTACGATAAAAACAAACTG
TTCGGCGTTACCACGCTGGACATCATCCGTTCCAATACCTTTGTGGCGGAGCTGAAAGGTAAATCGGCAACCGAG
GTGGAAGTCCCGGTCATTGGTGGTCACTCCGGGGTCACCATTCTGCCTTTACTGTCGCAGATCCCCGGCGTCAGC
TTTAGCGATCAGGAAATTGCCGACCTGACTAAACGTATTCAGAACGCCGGTACCGAAGTCGTGGAAGCGAAAGCG
GGCGGCGGGTCGGCGACCTTGTCGATG
>pgi68
GAGTCCAACGGTAAGTATGTTGACCGTAACGGCCACGCGGTAGACTACCAGACTGGCCCAATCATCTGGGGTGAG
CCGGGCACCAACGGTCAGCACGCGTTCTACCAGCTGATCCATCAGGGCACCAAAATGGTACCGTGCGATTTCATC
GCTCCGGCTATCACCCACAACCCGCTGTCTGACCACCATCAGAAACTGCTGTCTAACTTCTTCGCCCAGACCGAG
GCCCTGGCCTTTGGTAAATCCCGCGAAGTGGTTGAGCAGGAATATCGCGATCAGGGTAAAGACCCGGCGACCCTG
GAGCACGTGGTGCCGTTCAAAGTGTTCGAAGGTAACCGCCCGACTAACTCCATCCTGCTGCGTGAGATCACCCCG
TTCAGCCTCGGGGCGCTGATTGCCCTGTACGAGCACAAAATCTTCATCCAGGGCGCG
>phoE4
GTCGGCACCTCGTTAAGCTATGATTTCGGCGGCAGCGACTTCGCCGTCAGCGCAGCCTACACCAGCTCCGACCGT
ACCAACGATCAGAACCTGCTGGCCCGCGGCCAGGGTTCGAAAGCGGAAGCCTGGGCGACCGGCCTGAAATATGAC
GCCAACAATATCTACCTGGCGACCATGTACTCTGAAACCCGCAAGATGACCCCGATCAGCGGCGGCTTTGCCAAC
AAAGCGCAGAACTTTGAAGCGGTGGCGCAGTATCAGTTCGACTTCGGTCTGCGTCCGTCCCTCGGCTATGTGCTG
TCGAAAGGGAAGGATATCGAAGGGGTGGGAAGTGAGGATCTGGTTAACTACATTGACGTGGGCCTGACCTACTAC
TTCAACAAAAACATGAACGCCTTCGTGGATTACAAAATCAACCAG
> rpoB1
ACCCACTACGGTCGCGTATGTCCGATCGAAACGCCTGAAGGTCCGAACATCGGTCTGATTAACTCCCTGTCCGTG
TACGCGCAGACCAACGAATATGGCTTCCTTGAGACGCCGTATCGTAAAGTGACCAACGGTGTGGTTACTGACGAA
ATTCACTACCTGTCTGCTATCGAAGAAGGCAACTACGTTATCGCTCAGGCGAACTCCAACCTGGATGAAAACGGC
CACTTCGTAGAAGATCTGGTTACCTGCCGTAGCAAAGGCGAATCCAGCTTGTTCAGCCGCGACCAGGTTGACTAC
ATGGACGTATCCACCCAGCAGGTGGTATCCGTCGGTGCGTCCCTGATCCCGTTCCTGGAACACGATGACGCCAAC
CGTGCATTGATGGGTGCGAACATGCAACGTCAGGCGGTTCCGACTCTGCGCGCTGATAAGCCGCTGGTTGGTACC
GGTATGGAACGTGCTGTTGCCGTTGACTCCGGTGTTACTGCCGTGGCTAAA
>tonB39
ATGGTGGCGCCGGCCGATCTTGAGCCGCCTCCGGCGGCGCAGCCTGTCGTGGAGCCCGTTGTTGAACCCGAACCT
GAGCCGGAGCCAGAGGTAGCGCCTGAACCGCCGAAAGAGGCGCCGGTGGTGATCCATAAACCGGAACCTAAGCCG
AAGCCCAAACCTAAACCCAAGCCTAAGCCGGAGAAAAAGGTTGAACAGCCGAAGCGGGAAGTGAAGCCGGCAGCT
GAGCCGCGTCCGGCCTCGCCGTTTGAAAACAACAATACGGCGCCGGCGCGTACAGCGCCAAGCACCTCGACCGCA
GCGGCTAAACCCACCGTTACTGCTCCGAGCGGCCCGCGGGCGATCAGCCGCGTTCAGCCGTCCTATCCGGCGCGC
GCTCAGGCGCTGCGCATTGAAGGGACGGTACGGGTGAAG

ST101

>gapA2

AACCTGAAGTGGGACGAAGTTGGTGTTGACGTTGTTGCTGAAGCAACCGGTATCTTCCTGACCGACGAAACCGCT
CGTAAACACATCACCGCTGGCGCGAAAAAAGTCGTTCTGACTGGCCCGTCCAAAGACAACACTCCGATGTTCGTT
CGCGGCGCTAACTTCGACGCTTACGCTGGCCAGGACATCGTTTCCAACGCTTCCTGCACCACCAACTGCCTGGCG
CCGCTGGCTAAAGTTATCAACGACAACTTCGGTATCGTTGAAGGCCTGATGACCACCGTCCACGCTACCACCGCT
ACTCAGAAAACCGTTGATGGCCCGTCTCACAAAGACTGGCGCGGCGGCCGCGGCGCAGCTCAGAACATCATCCCG
TCCTCTACCGGCGCTGCTAAAGCAGTAGGTAAAGTACTGCCAGAACTGAACGGCAAACTGACCGGTATGGCGTTC
>infB6
GGCATGATCACCTTCCTGGATACCCCGGGCCACGCCGCGTTTACCTCCATGCGTGCTCGTGGCGCGCAGGCGACG
GATATCGTGGTTCTGGTGGTGGCGGCAGACGACGGCGTGATGCCGCAGACTATCGAAGCTATCCAGCACGCTAAA
GCGGCGCAGGTACCGGTGGTAGTGGCGGTGAACAAGATCGATAAGCCAGAAGCCGATCCGGATCGCGTGAAGAAC
GAACTGTCCCAGTACGGCATCCTGCCGGAAGAGTGGGGCGGCGAGAGCCAGTTCGTCCACGTTTCCGCGAAAGCC
GGTACCGGCATCGACGAC
>mdh1
GGCGCGGATGTAGTGCTGATCTCCGCGGGCGTGGCGCGTAAGCCCGGCATGGATCGTTCCGACCTGTTTAATGTG
AATGCGGGTATCGTGAAGAACCTCGTGCAGCAGATTGCCAAAACCTGCCCGCAGGCCTGCATCGGCATTATCACC
AACCCGGTGAATACCACCGTGGCTATCGCCGCCGAAGTACTGAAAAAAGCCGGCGTGTACGATAAAAACAAACTG
TTCGGCGTTACCACGCTGGACATCATCCGTTCCAATACCTTTGTGGCGGAGCTGAAAGGTAAATCGGCAACCGAG
GTGGAAGTCCCGGTCATTGGTGGTCACTCCGGGGTCACCATTCTGCCTTTACTGTCGCAGATCCCCGGCGTCAGC
TTTAGCGATCAGGAAATTGCCGACCTGACTAAACGTATTCAGAACGCCGGTACCGAAGTCGTGGAAGCGAAAGCG
GGCGGCGGGTCGGCGACCTTGTCGATG
>pgi5
GAGTCCAACGGTAAGTATGTTGACCGTAACGGCCACGCGGTAGACTACCAGACTGGCCCAATCATCTGGGGGGAG
CCGGGCACCAACGGTCAGCACGCGTTCTACCAGCTGATCCACCAGGGCACCAAAATGGTACCGTGCGATTTCATC
GCTCCGGCTATCACCCACAACCCGCTGTCTGACCACCATCAGAAACTGCTGTCTAACTTCTTCGCCCAGACCGAG
GCCCTGGCCTTTGGTAAATCCCGCGAAGTGGTTGAGCAGGAATATCGCGATCAGGGTAAAGACCCGGCGACCCTG
AGCACGTGGTGCCGTTCAAAGTGTTCGAAGGTAACCGCCCGACTAACTCCATCCTGCTGCGTGAGATCACCCCGT
TCAGCCTCGGGGCGCTGATTGCCCTGTACGAGCACAAAATCTTCACCCAGGGCGCG
>phoE4
GTCGGCACCTCGTTAAGCTATGATTTCGGCGGCAGCGACTTCGCCGTCAGCGCAGCCTACACCAGCTCCGACCGT
ACCAACGATCAGAACCTGCTGGCCCGCGGCCAGGGTTCGAAAGCGGAAGCCTGGGCGACCGGCCTGAAATATGAC
GCCAACAATATCTACCTGGCGACCATGTACTCTGAAACCCGCAAGATGACCCCGATCAGCGGCGGCTTTGCCAAC
AAAGCGCAGAACTTTGAAGCGGTGGCGCAGTATCAGTTCGACTTCGGTCTGCGTCCGTCCCTCGGCTATGTGCTG
TCGAAAGGGAAGGATATCGAAGGGGTGGGAAGTGAGGATCTGGTTAACTACATTGACGTGGGCCTGACCTACTAC
TTCAACAAAAACATGAACGCCTTCGTGGATTACAAAATCAACCAG
>rpoB1
ATGGACGTATCCACCCAGCAGGTGGTATCCGTCGGTGCGTCCCTGATCCCGTTCCTGGAACACGATGACGCCAAC
CGTGCATTGATGGGTGCGAACATGCAACGTCAGGCGGTTCCGACTCTGCGCGCTGATAAGCCGCTGGTTGGTACC
GGTATGGAACGTGCTGTTGCCGTTGACTCCGGTGTTACTGCCGTGGCTAAA
>tonB6
ATGGTGGCGCCGGCCGATCTTGAGCCGCCTCCGGCGGCGCAGCCTGTCGTGGAGCCCGTTGTTGAACCCGAACCT
GAGCCGGAGCCAGAGGTAGTGCCTGAACCGCCGAAAGAGGCGCCGGTGGTGATCCATAAACCGGAACCTAAGCCG
AAGCCCAAACCTAAACCCAAGCCTAAGCCGGAGAAAAAGGTTGAACAGCCGAAGCGGGAAGTGAAGCCGGCAGCA
GAGCCGCGTCCGGCCTCGCCGTTTGAAAACAACAATACGGCGCCGGCGCGTACAGCGCCAAGTACCTCGACCGCA
GCGGCTAAACCCACCGTTACTGCTCCGAGCGGCCCGCGGGCGATCAGCCGCGTTCAGCCGTCCTATCCAGCGCGC
GCTCAGGCGCTGCGCATAGAAGGGACGGTACGGGTGAAG
